# Genome-wide identification and characterization of FORMIN gene family in cotton (*Gossypium hirsutum* L.) and their expression profiles in response to multiple abiotic stress treatments

**DOI:** 10.1371/journal.pone.0319176

**Published:** 2025-03-03

**Authors:** Suronjeet Kumar Paul, Md Shohel Ul Islam, Nasrin Akter, Fatema Tuz Zohra, Shuraya Beente Rashid, Md. Shakil Ahmed, Shaikh Mizanur Rahman, Md. Abdur Rauf Sarkar

**Affiliations:** 1 Laboratory of Functional Genomics and Proteomics, Department of Genetic Engineering and Biotechnology, Faculty of Biological Science and Technology, Jashore University of Science and Technology, Jashore, Bangladesh; 2 Department of Genetic Engineering and Biotechnology, Faculty of Biological Sciences, University of Rajshahi, Rajshahi, Bangladesh; 3 Department of Biochemistry and Molecular Biology, Faculty of Science, University of Rajshahi, Rajshahi, Bangladesh; PLOS ONE, UNITED KINGDOM OF GREAT BRITAIN AND NORTHERN IRELAND

## Abstract

FORMIN proteins distinguished by FH2 domain, are conserved throughout evolution and widely distributed in eukaryotic organisms. These proteins interact with various signaling molecules and cytoskeletal proteins, playing crucial roles in both biotic and abiotic stress responses. However, the functions of FORMINs in cotton (*Gossypium hirsutum* L.) remain uncovered. In this study, 46 FORMIN genes in *G. hirsutum* (referred to as *GhFH*) were systematically identified. The gene structures, conserved domains, and motifs of these *GhFH* genes were thoroughly explored. Phylogenetic and structural analysis classified these 46 *GhFH* genes into five distinct groups. *In silico* subcellular localization, prediction suggested that *GhFH* genes are distributed across various cellular compartments, including the nucleus, extracellular space, cytoplasm, mitochondria, cytoskeleton, plasma membrane, endoplasmic reticulum, and chloroplasts. Evolutionary and functional diversification analyses, based on on-synonymous (*Ka*) and synonymous (*Ks*) ratios and gene duplication events, indicated that *GhFH* genes have evolved under purifying selection. The analysis of *cis*-acting elements suggested that *GhFH* genes may be involved in plant growth, hormone regulation, light response, and stress response. Results from transcriptional factors TFs and gene ontology analysis indicate that FORMIN proteins regulate cell wall structure and cytoskeleton dynamics by reacting to hormone signals associated with environmental stress. Additionally, 45 putative ghr-miRNAs were identified from 32 families targeting 33 *GhFH* genes. Expression analysis revealed that *GhFH1*, *GhFH10*, *GhFH20*, *GhFH24*, and *GhFH30* exhibited the highest levels of expression under red, blue, and white light conditions. Further, *GhFH9*, *GhFH20*, and *GhFH30* displayed higher expression levels under heat stress, while *GhFH20* and *GhFH30* showed increased expression under salt stress compared to controls. The result suggests that *GhFH20* and *GhFH30* genes could play significant roles in the development of *G. hirsutum* under heat and salt stresses. Overall these findings enhance our understanding of the biological functions of the cotton FORMIN family, offering prospects for developing stress-resistant cotton varieties through manipulation of *GhFH* gene expression.

## 1. Introduction

Light serves as a crucial energy source for photosynthesis and acts as a key signal in plant growth and development [1]. It primarily influences plant growth by regulating the activity of photosynthetic genes, which in turn contribute to the production of carbohydrates and other important secondary compounds in plants [2,3]. Plants possess photoreceptors such as phototropins, phytochromes, and cryptochromes which absorb light and initiate signaling pathways that affect plant physiology [4]. Due to their immobility, plants are exposed to various environmental stresses, including heavy metals, high salinity, drought, nutrient shortages, varying light levels, pesticide pollution, and extreme temperature conditions [5]. To adapt to these external conditions plants undergo rapid morphological changes in roots, leaves, and pollen, driven by cytoskeleton dynamics [6]. FORMINs are known to regulate actin cytoskeleton dynamics by facilitating actin polymerization [7]. These proteins enhance actin polymerization by stimulating filament nucleation and elongation [8,9]. In pants, three key FORMIN domains have been identified: FORMIN Homology 1 (FH1), FORMIN Homology 2 (FH2), and FORMIN Homology 3 (FH3) [10]. Among these, the FH2 domain is crucial for polymerase function and serves as a specific marker for identifying gene families [11]. Plants FORMINs regulate actin filaments and control the dynamic remodeling actin cytoskeleton enabling plant cells to change their shapes and manage the cellular structure of plant tissues and organs [12]. FORMINs have been implicated in pathogen resistance and play a crucial role in male fertility in wheat (*Triticum aestivum*) [9,13]. The FH2 domain has been observed to influence actin polymerization dynamics through various mechanisms, including altering the rate of filament elongation and depolymerization, enhancing *de no*vo filament nucleation, and preventing filament barbed-end capping by capping proteins [14].

Numerous FORMIN genes have been identified in various plant species, and comprehensive genome-wide analyses have been conducted in recent studies, such as 17 genes in rice (*Oryza sativa*) [15], 22 genes in *Arabidopsis* (*Arabidopsis thaliana*) [16], 25 genes in wheat (*Triticum aestivum*) [9]. In *Arabidopsis*, the functions of specific genes such as *AtFH1*, *AtFH8*, *AtFH6*, and *AtFH5* have been studied *in vivo*. For example, *AtFH1* influences pollen tube elongation, a polar cell growth process dependent on a precisely controlled actin cytoskeleton [16]. The fusion protein AtFH5-GFP exhibits a distinct concentration within the cell plate, which is crucial for cell division [17]. *AtFH8* affects root and root hair development by modifying the distribution of the actin cytoskeleton [18]. In rice, FORMIN gene expression patterns have been shown to respond to both drought and cadmium (Cd) stress [15].

Cotton is a significant crop, well known for its high-quality natural fibers that are essential to the global textile industry [19]. Additionally, cotton serves as a source of edible oil and plant proteins [20]. It is the largest genus in the Gossypieae tribe, with over 50 species, among which, *G. hirsutum* is the most widely cultivated. Native to southern Florida, the Caribbean, Mexico, and Central America [21]. *G. hirsutum* is distinguished by its broad adaptability and high yield, contributing to over 95% of global cotton production [22]. The species originated from a significant polyploidization event around 1–2 million years ago, resulting from the merging of the A and D genomes from *G. arboreum* and *G. raimondii*, respectively [23]. Despite the importance of FORMIN proteins, their function has not been studied in *G. hirsutum*. Conducting wet lab experiments, to identify the FORMIN gene family, and analyze its expression is costly in terms of labor, time, and the need for well-equipped laboratories. In this study, we have systematically identified and characterized the members of the FORMIN gene family in *G. hirsutum* using integrated bioinformatics approaches to gain a better understanding of their functional roles under various physiological conditions. Each of *GhFH* members was further analyzed to determine their physiochemical properties, phylogenetic relationship, conserved domain, motifs, gene structures, *Ka*/*Ks* ratio, collinearity, synteny analysis, sub-cellular localization, transcription factors, protein-protein interactions, gene ontology, and *cis*-acting elements. Additionally, transcript profiling of identified *GhFH* members was conducted in response to three different light conditions and various abiotic stress conditions using RNA-seq data. Our study provides a foundation for further exploration of the FORMIN protein family in *G. hirsutum* and contributes to improving present cotton cultivar.

## 2. Materials and methods

### 2.1. Database search and retrieval of FORMIN protein sequences in *G. hirsutum* genome

The FH2 DNA-binding domains from *A. thaliana* were used to retrieve FH2 gene-encoding proteins in the *G. hirsutum* v3.1 genome from Phytozome v13 (https://phytozome-next.jgi.doe.gov/) using BLASTp (Protein-basic local alignment search tool) [24], with an expected (E) threshold value of −1, a comparison matrix (BLOSUM62), and other default parameters. The conserved FH2 domain was then identified in the retrieved amino acid sequences using the NCBI CDD (Conserved Domain Database) (https://www.ncbi.nlm.nih.gov/Structure/cdd/wrpsb.cgi) [25] SMART (Simple Modular Architecture Research Tool, (http://smart.embl-heidelberg.de/) [26] and the pfam database (http://pfam.xfam.org/) [27] at default settings. Redundant protein sequences that did not contain the FH-conserved domain were excluded from the list of candidates (S1 Data).

### 2.2. Determination of physio-chemical properties

The ProtParam online tool (http://web.expasy.org/protparam/) was used to determine the physicochemical properties of GhFH proteins, including their amino acid residue count, molecular weight, isoelectric point (pI), instability index, aliphatic index, and grand average of hydropathicity (GRAVY) [28].

### 2.3. Phylogenetic tree analysis

Protein sequences from *Gossypium hirsutum*, *Arabidopsis thaliana*, *Medicago truncatula*, *Oryza sativa*, and *Zea mays* were used to construct a phylogenetic tree (S2 Data). The MEGA11 software [29], was employed to construct the phylogenetic tree, and precise sequence alignment was performed using, the ClustalW program [30,31]. The maximum likelihood (ML) method was applied in MEGA11 software with default parameters, except for a bootstrap value of 1000 and Pearson correction. The final tree was then uploaded to iTOL v6 (https://itol.embl.de/) [32] for enhanced visual representation.

### 2.4. Analysis of gene structure

The gene structure of *GhFHs* was determined by retrieving genomic DNA and CDS sequences in FASTA format from Phytozome v13 (S3 and S4 Data). The Gene Structure Display Server (GSDS v2.0) (http://gsds.cbi.pku.edu.cn/) [33] was used for the analysis.

### 2.5. Conserved domain and motif analysis

The NCBI Conserved Domain Search (https://www.ncbi.nlm.nih.gov/Structure/cdd/wrpsb.cgi) was utilized to identify the typical conserved FH2 domain (pfam02181) with results displayed using the DOG2.0 software [34]. Structural motifs of GhFH protein sequences were explored using the MEME-suite tools (https://meme-suite.org/meme/meme_5.5.3/tools/meme) [35] with a maximum of 20 motifs, selected, and visualized using the TBtools software-v1.116 [36].

### 2.6. Prediction of the subcellular localization of GhFH proteins

The *in silico* subcellular localization of GhFH proteins was predicted using the WoLF PSORT online tool (https://wolfpsort.hgc.jp/) [37]. The predicted protein signals for each *GhFH* gene were visualized using RStiduo 4.2.1 software [38] with the following libraries: scales, extrafont, ggplot2, and reshape.

### 2.7. *Cis*-acting regulatory elements (CAREs) analysis of *GhFH* promoters

The 2000 bp upstream promoter region of each *GhFH* gene sequence was obtained from the Phytozome v13 database (S5 Data) for investigation of CAREs. The PlantCARE online tool (https://bioinformatics.psb.ugent.be/webtools/plantcare/html/) [39] was used for CAREs analysis, with results visualized using RStiduo 4.2.1 software with the following libraries: scales, extrafont, ggplot2, and reshape.

### 2.8. *Ka/Ks* analysis

*Ks* and *Ka* values along with their substitution ratios for the *GhFH* gene family were calculated using the *Ka/Ks* calculation tool within TBtools software-v1.116. The molecular evolution rates for each set of paralogous genes were determined based on the *Ka/Ks* ratios. The formula, T = *Ks*/2X, where X = 6.56 × 10^−9^) [40], was used to calculate the duplication events and time of divergence (measured in million years ago, MYA = 10^−6^) for the *GhFH* gene. Gene duplication predictions were performed using MCScanX within TBtools software-v1.116.

### 2.9. Gene ontology (GO) analysis

GO analysis was conducted to identify the functions of predicted *GhFH* genes in *G. hirsutum* using the Plant Transcription Factor Database (PlantTFDB, http://planttfdb.cbi.pku.edu.cn//) [41], with visualized using the online tool ChiPlot (https://www.chiplot.online/).

### 2.10. Collinearity and synteny analysis of *GhFH* gene family

Collinearity and synteny relationships within the cotton genome, as well as the FORMIN genes rice, maize, and *Arabidopsis* were visualized and analyzed using TBtools version-v1.116.

### 2.11. Transcription factor (TFs) analysis and regulatory network of *GhFH* genes

The online tool PlantTFDB4.0 (http://planttfdb.cbi.pku.edu.cn//) [41] was utilized to predict TFs associated with the candidate *GhFH* genes. The results were visualized using the online tool ChiPlot (https://www.chiplot.online/). The interaction network between the *GhFH* genes and TFs was constructed and illustrated using Cytoscape 3.9.1 software [42].

### 2.12. Protein-protein interaction (PPI) analysis

The STRING version 12.0 online program (https://string-db.org/) was used to predict and design the PPI network for GhFH proteins, utilizing homologous proteins from *Arabidpsois*. The STRING tool was configured with specific parameters: network type-full STRING network, network edges meaning-evidence, a minimum required interaction score set at a medium confidence parameter (0.4), and a maximum display of no more than 10 interactions.

### 2.13. Identification of miRNAs targeting *GhFH* genes

CDS sequences of *GhFH* genes were uploaded to the online psRNATarget Server18 (https://www.zhaolab.org/psRNATarget/analysis?function=2) [43] with default parameters to predict putative miRNAs targeting *GhFH* genes. The interaction network between the predicted miRNAs and *GhFH* target genes was generated and visualized using Cytoscape software version 3.9.1

### 2.14. Expression analysis of *GhFH* genes in different light conditions

RNA-Seq data for *G. hirsutum* were obtained from the NCBI Sequence Read Archive (SRA) database [44] using the accession number SRA: PRJNA765172 [45]. Quality control and trimming of the RNA-Seq data were performed using the Trimmomatic v0.32 package [46]. The data then aligned to the *G. hirsutum* reference genome with STAR packages v2.7.11b [47]. Sequence alignment map (SAM) files were converted to binary alignment map (BAM) format and sorted using Samtools v1.20 [48]. FPKM (Fragments Per Kilobase of transcript per Million mapped reads) values were calculated with the RSEM package v1.1.17 [49]. Due to significant variations in FPKM values across different *G. hirsutum* tissue samples, the values were log2 transformed. Heatmaps to visualize the expression profiles of *GhFH* genes in different light conditions were constructed using TB-Tools software v1.116.

### 2.15. Expression analysis of *GhFH* genes in various tissues and abiotic stresses

RNA-Seq raw reads for *G. hirsutum* were downloaded from the NCBI SRA database with the accession number SRA: PRJNA248163 [50]. Quality controlled and filtering of the RNA-Seq raw reads were performed using trimmomatic package version 0.32 and the reads were mapped to the *G. hirsutum* reference genome using the Bowtie2 package [51]. SAM files were transformed to BAM format and sorted using Samtools packages version 1.20. FPKM values were calculated using the RSEM package v1.1.17. Due to large differences in FPKM values among different tissues of *G. hirsutum*, the FPKM values were transformed to log2. Heatmaps to visualize the expression profiles of *GhFH* genes in different tissues and stress conditions were constructed using TB-Tools software v1.116.

## 3. Results

### 3.1. Analysis of physicochemical properties of GhFH

The FORMIN gene family is recognized as essential for the growth, function, and development of plants. By regulating both the microtubule and actin cytoskeleton, FORMINs contribute to vital cellular processes including cytokinesis, cell polarity, and cell migration [17,52]. The presence of the typical FH2 domain is used to identify the FORMIN family in plants [11]. In the *G. hirsutum* genome, 46 FORMIN genes were identified and designated as *GhFH1*-*GhFH46*. The physiochemical analysis of the protein sequences of *GhFH* genes revealed notable differences in the chemical and physical properties among the members of the GhFH family (Table 1). The amino acid count in GhFH proteins ranged from 402 to 4461. Significant variations in molecular weights were observed, ranging from 101138.61 kDa (GhFH34) to 375608.96 kDa (GhFH43). Furthermore, the isoelectric points (pI) of these 46 GhFH proteins exhibited diversity, ranging from 4.69 to 5.32. The instability index revealed that the GhFH18 protein had the lowest value at 25.16, while the GhFH37 protein showed the highest instability index value at 72.58. The aliphatic index values ranged from 23.95 to 33.58, with an average value of 29.09. Additionally, all GhFH proteins exhibited an average hydropathicity of less than 1, except for GhFH37, which scored 1.08.

**Table 1:** List of 46 *GhFH* genes and their basic physiochemical characterization.

SI	Gene identifier	Gene name	Size (aa)	Mass (kDa)	pI	Instability index	Aliphatic index	GRAVY
1	Gohir.A02G007500	*GhFH1*	2799	237323.45	4.82	49.77	27.58	0.917
2	Gohir.A03G143800	*GhFH2*	2376	196496.88	4.89	42.06	30.01	0.845
3	Gohir.A04G022700	*GhFH3*	2778	231720.54	4.86	43.76	29.59	0.831
4	Gohir.A04G089001	*GhFH4*	444	37134.29	5.32	25.36	29.73	0.493
5	Gohir.A05G059300	*GhFH5*	3864	325089.78	4.78	45.98	28.67	0.815
6	Gohir.A05G415300	*GhFH6*	2037	167794.83	4.93	44.33	30.09	0.811
7	Gohir.A07G070200	*GhFH7*	3702	308059.04	4.80	45.28	27.93	0.776
8	Gohir.A07G175778	*GhFH8*	444	37148.32	5.32	25.55	29.95	0.498
9	Gohir.A07G205332	*GhFH9*	639	52886.37	5.26	26.69	29.42	0.503
10	Gohir.A08G020008	*GhFH10*	2844	237904.89	4.86	44.84	29.01	0.798
11	Gohir.A08G228101	*GhFH11*	3765	318539.36	4.77	45.53	26.75	0.836
12	Gohir.A09G242700	*GhFH12*	4281	358550.88	4.75	50.02	28.15	0.825
13	Gohir.A10G146300	*GhFH13*	2742	231853.47	4.82	48.62	26.55	0.879
14	Gohir.A11G032500	*GhFH14*	1890	157618.91	4.91	55.96	28.10	0.904
15	Gohir.A11G144300	*GhFH15*	3357	282986.80	4.79	43.17	25.89	0.820
16	Gohir.A11G215800	*GhFH16*	2772	229193.24	4.86	46.65	28.93	0.817
17	Gohir.A12G076200	*GhFH17*	2697	222787.26	4.86	44.49	29.55	0.834
18	Gohir.A12G082833	*GhFH18*	444	37092.27	5.32	25.16	29.73	0.500
19	Gohir.A12G135200	*GhFH19*	2466	202452.31	4.90	42.26	30.78	0.811
20	Gohir.A12G179700	*GhFH20*	4149	348141.31	4.74	48.73	27.26	0.865
21	Gohir.A13G108800	*GhFH21*	2424	200670.50	4.91	43.42	33.42	0.842
22	Gohir.A13G141900	*GhFH22*	2844	236172.28	4.86	43.69	29.29	0.787
23	Gohir.A13G184401	*GhFH23*	3543	295168.92	4.80	43.42	29.16	0.828
24	Gohir.D02G007300	*GhFH24*	2796	237260.26	4.82	50.11	27.58	0.912
25	Gohir.D02G110350	*GhFH25*	402	33565.70	5.25	36.66	33.58	0.871
26	Gohir.D02G166900	*GhFH26*	2409	199719.82	4.88	42.35	29.80	0.853
27	Gohir.D04G000900	*GhFH27*	2046	168576.68	4.93	45.12	29.96	0.808
28	Gohir.D05G061366	*GhFH28*	1185	96453.43	5.09	37.45	31.81	0.715
29	Gohir.D05G364800	*GhFH29*	2784	232389.07	4.86	41.91	29.13	0.816
30	Gohir.D06G097600	*GhFH30*	3813	319654.53	4.79	45.91	28.93	0.819
31	Gohir.D07G074800	*GhFH31*	3879	323119.72	4.78	45.42	28.44	0.802
32	Gohir.D07G094100	*GhFH32*	3111	261853.51	4.81	48.85	28.22	0.871
33	Gohir.D08G030700	*GhFH33*	2856	239230.54	4.85	44.01	28.68	0.798
34	Gohir.D08G248366	*GhFH34*	1239	101138.61	5.08	32.93	31.96	0.714
35	Gohir.D09G243400	*GhFH35*	3456	290291.18	4.78	53.75	28.12	0.886
36	Gohir.D10G120700	*GhFH36*	2757	233618.67	4.82	49.47	27.02	0.890
37	Gohir.D11G035801	*GhFH37*	3867	341003.22	4.69	72.58	23.95	1.080
38	Gohir.D11G150700	*GhFH38*	3306	278574.76	4.80	44.20	25.86	0.818
39	Gohir.D11G207500	*GhFH39*	2772	229257.48	4.86	45.77	29.08	0.824
40	Gohir.D11G277200	*GhFH40*	2613	216470.99	4.89	40.47	31.19	0.805
41	Gohir.D12G074600	*GhFH41*	2694	222436.00	4.86	44.62	29.92	0.844
42	Gohir.D12G139300	*GhFH42*	2484	204158.41	4.89	42.76	30.68	0.816
43	Gohir.D12G182300	*GhFH43*	4461	375608.96	4.72	49.42	26.97	0.869
44	Gohir.D13G111700	*GhFH44*	2418	200135.98	4.91	42.58	33.58	0.847
45	Gohir.D13G146600	*GhFH45*	2880	239035.56	4.86	43.70	29.24	0.790
46	Gohir.D13G190601	*GhFH46*	3540	295031.65	4.80	43.10	29.18	0.824

MW, molecular weight (kDa); aa, number of amino acids; pI, theoretical isoelectric point; GRAVY, grand average of hydropathicity. Instability index (<40, the protein is stable >40, the protein is unstable) [53].

### 3.2. Analysis of phylogenetic tree

**Fig 1:**
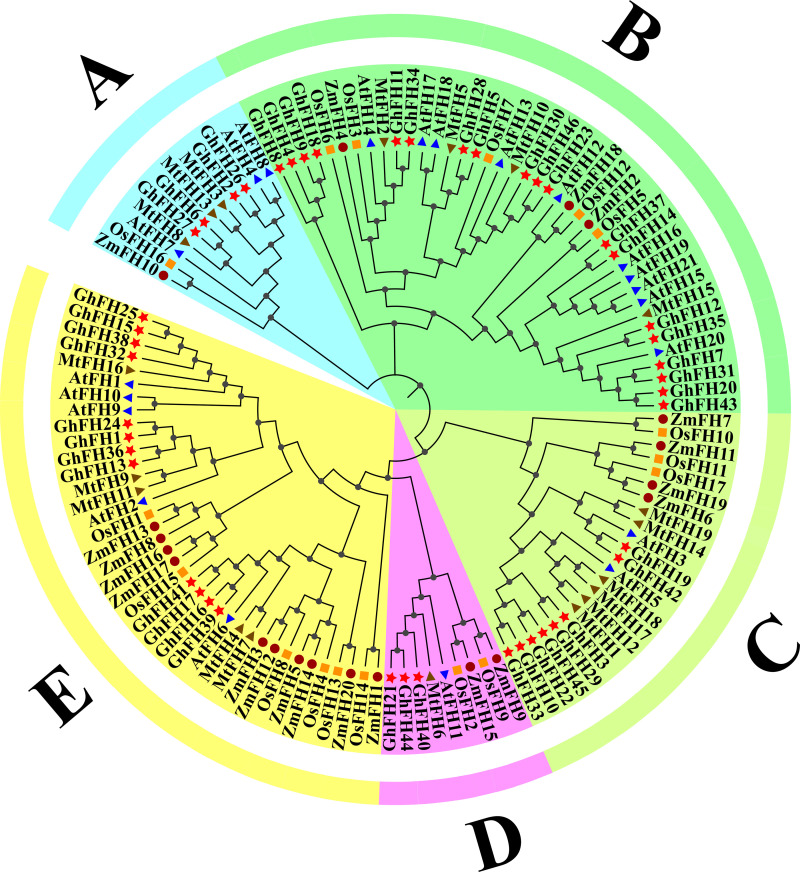
Phylogenetic tree analysis of FORMIN family members. Phylogenetic tree displaying the evolutionary relationships of FORMIN proteins based on the FH2 domain from *G. hirsutum, A. thaliana, M. truncatula, O. sativa,* and *Z. mays.* All the FH members were divided into 5 groups and presented in different colors. The red star represented GhFH proteins, the blue triangle represented AtFH proteins, the brown triangle represented MtFH proteins, the orange square represented OsFH proteins and the pink circle represented ZmFH proteins.

To explore the conservation and evolutionary relationships of FORMIN proteins across different species, a phylogenetic tree was constructed using 46 proteins from *G. hirsutum*, 21 proteins from *A. thaliana*, 19 proteins from *M. truncatula*, 17 proteins from *O. sativa*, and 20 proteins from *Z. mays* (Fig 1). Proteins located closer together within a cluster on the phylogenetic tree, exhibit a higher degree of functional similarity [54]. The phylogenetic analysis of the 46 *G. hirsutum* proteins separated them into five distinct groups (A, B, C, D, and E). Group B contained the highest number of GhFH proteins with 19, while group D contained the lowest number of proteins with 3. Groups A, C, and E contained 4, 8, and 12 GhFH proteins, respectively (S6 Data). According to the phylogenetic tree GhFH6, GhFH2, GhFH3, and GhFH13 from G. *hirsutum* were closely related to MtFH13, MtFH3, MtFH1, and MtFH9 from M. *truncatula’s*, respectively. Additionally, ZmFH10 exhibited a close relation with OsFH16, ZmFH18 with OsFH12, ZmFH7 with OsFH10, ZmFH11 with OsFH11, ZmFH9 with OsFH9, ZmFH20 with OsFH13, and ZmFH15 with OsFH2..

### 3.3. Analysis of *GhFH* gene structure

**Fig 2:**
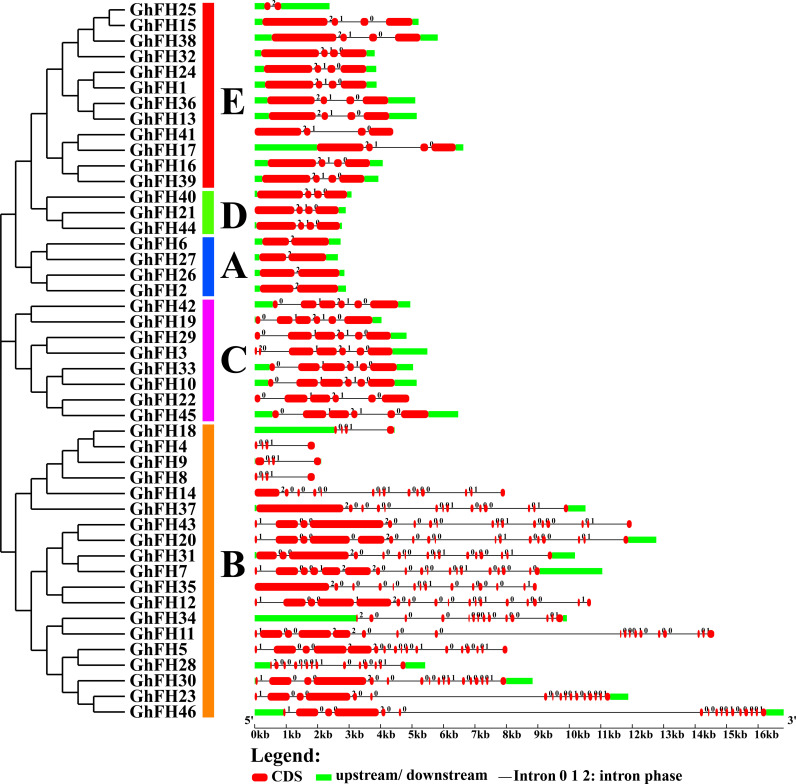
Gene structure of *GhFH* genes. Gene structure analyses for *GhFH* genes were carried out using the Gene Structure Display Server (GSDS 2.0, http://gsds.cbi.pku.edu.cn/index.php). The lengths of exons and introns for each *GhFH* gene are demonstrated proportionally. Gene groups are categorized and colored based on their phylogenetic relationships. For all *GhFH* genes, black lines represent introns, red-bold lines represent exons, and light-green lines represent 5’ and 3’ untranslated regions (UTR). The structure of each *GhFH* gene exon/intron is displayed proportionally according to the scale mentioned.

The ability to encode proteins and perform cellular function is determined by its structure [55]. The gene structure analysis of 46 *GhFH* genes revealed that all genes contained exons and introns but lacked upstream/downstream regions (Fig 2). The analysis showed that *GhFH46* had the longest gene length among the studied genes, while *GhFH4* and *GhFH8* had the shortest gene lengths. *GhFH7* and *GhFH11* had the highest number of exon sequences with 18 and intron sequences with 17 (S7 Data). Interestingly, *GhFH4* and *GhFH8* genes shared the same structure and length of approximately 2.0 kb. The genes in group A (*GhFH2*, *GhFH26*, *GhFH27*, *GhFH6*) were found to have similar structure.

### 3.4. Analysis of conserved domains in GhFH

**Fig 3:**
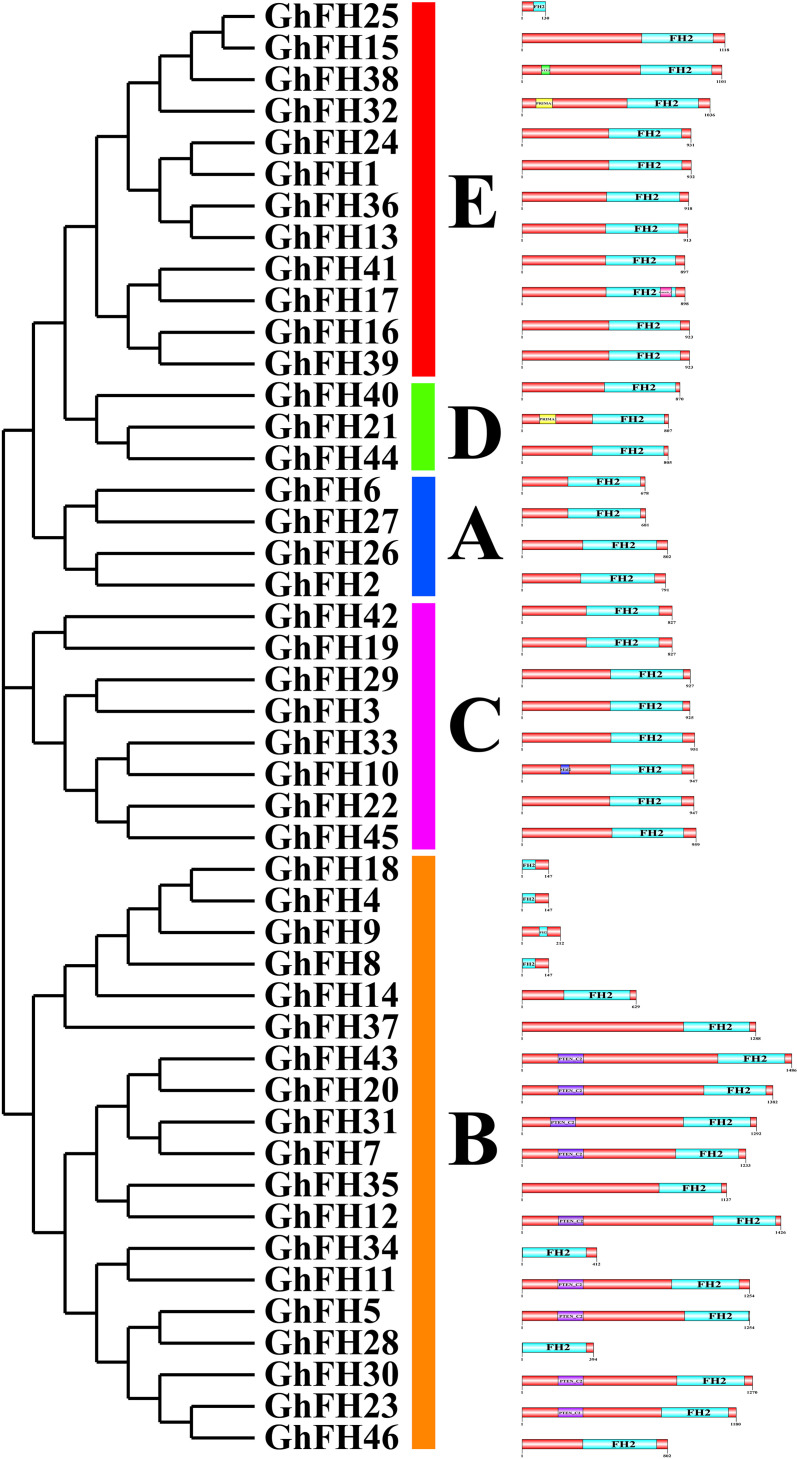
Conserved domains of GhFH proteins. The positions of each conserved domain are demonstrated in differently colored boxes, with the domain names.

Domain unravels the structure, function, and evolution of proteins [56]. In our analysis, we mainly focused on the typical FH2 domain along with PTEN_C2, PRIMA1, STE3, Remorin_c, and Mid2, respectively (Fig 3). The proteins of group A were found to contain only the FH2 domain.

### 3.5. Analysis of GhFH motifs

**Fig 4:**
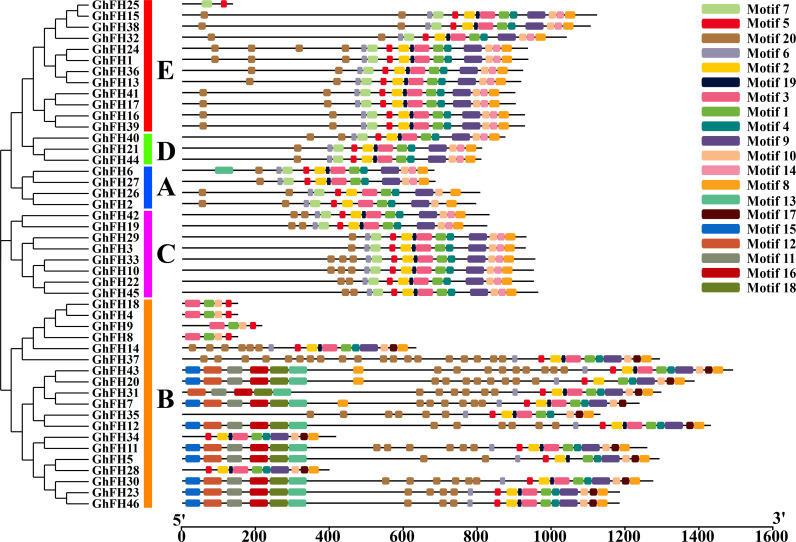
Distribution of conserved motifs of GhFH proteins. The identification of conserved motifs in GhFH proteins was carried out using the Multiple EM for Motif Elicitation (MEME) (https://meme-suite.org/meme/tools/meme) tool, with a maximum of 20 motifs selected. Each motif is represented by a specific-colored box aligned on the right side of the figure. Different colors indicate individual motifs identified within each protein domain.

Motifs serve as representations of structural components, active sites, transcription binding sites, and splice junctions within genetic sequences [57]. In our analysis, 20 different motifs present in the GhFH peptide sequence were identified (S1 Fig). Closely related proteins within each phylogenetic group were found to share identical motif compositions (Fig 4). GhFH25 was found to contain only two motifs (Motif7, Motif5). Additionally, GhFH8, GhFH9, GhFH4, GhFH18, GhFH34, and GhFH28 exhibited different motifs from others present in group B, which may have some functional implications.

### 3.6. Analysis of subcellular localization of GhFH proteins

**Fig 5:**
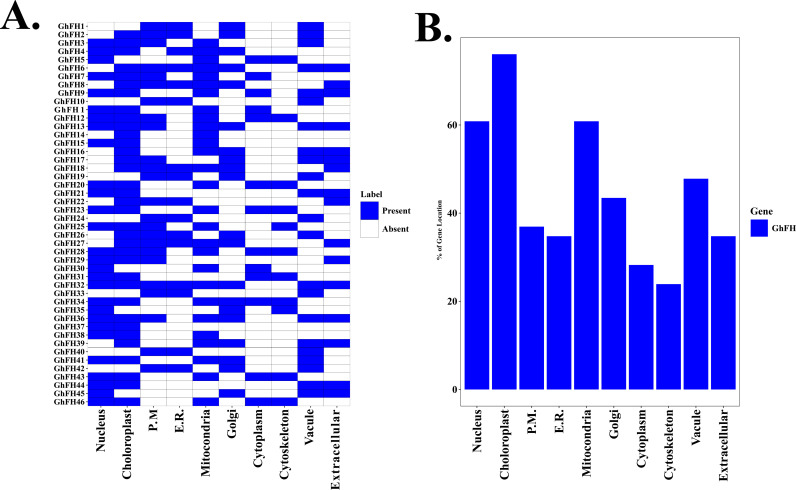
Subcellular localization of GhFH proteins. The subcellular distribution of GhFH proteins is shown in a heatmap. A. The relevant cellular organelles are displayed at the bottom of the heatmap, and the names of each GhFH protein are displayed on the left side. The presence of protein signals corresponding to the genes is shown by a blue color on the heatmap. B. A bar diagram illustrates the percentage distribution of the GhFH protein signal across different cellular organelles. The percentages of protein signals that exist in various cellular organelles are displayed on the left side.

The cellular compartment in which a protein is present strongly influences its functions and actions [58]. In this study, GhFH proteins were mainly located in three major organelles: chloroplasts, mitochondria, and the nucleus. Almost all GhFH proteins are localized in chloroplasts, except for GhFH1, GhFH5, GhFH10, GhFH19, GhFH24, GhFH30, GhFH33, GhFH35, GhFH40, GhFH42, and GhFH45 (Fig 5A). This analysis revealed that 76.5% of GhFH proteins were present in chloroplasts, 60.84% within the nucleus, 36.94% in the plasma membrane, 34.77% in the endoplasmic reticulum (E.R), 60.84% in mitochondria, 43.46% in the Golgi, 28.25% in the cytoplasm, 23.90% in the cytoskeleton, 47.81% in vacuoles, and 34.77% in the extracellular membrane (Fig 5B), providing a complete illustration of their distribution.

### 3.7. Analysis of *cis*-acting regulatory elements (CAREs) of *GhFH* promoters

**Fig 6:**
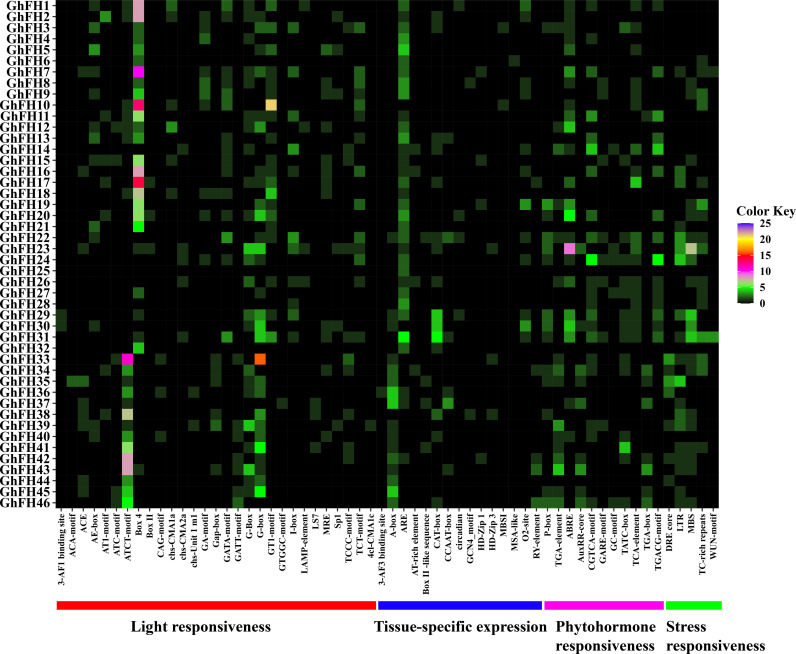
The distribution of CAREs in the 2.0 kb promoter region of *GhFH* genes is represented by a heatmap. The names of each *GhFH* gene are shown on the left side of the heatmap. The number of putative CAREs for each *GhFH* gene is displayed on the bottom of the heatmap with a color scale (0–25) on the right side of the heatmap. Functions associated with CAREs of the corresponding genes, such as light responsiveness, tissue-specific expression, phytohormone responsiveness, and stress responsiveness, are indicated by bold lines in red, blue, pink, and green at the bottom of the heatmap, respectively.

The 2000 base pairs upstream of the 5′ end of the 46 *GhFH* genes were analyzed to understand their possible regulatory mechanisms. A total of 62 CAREs that play pivotal roles in light response, phytohormone sensitivity, tissue-specific expression, and stress responsiveness were identified in the *G. hirsutum genome* (Fig 6). Among these CAREs, 5 were stress-responsiveness, including DREs, MBSs, LTRs, WUN motif-containing elements, and TC-rich repeats while 17 were related to tissue-specific expression. Notable tissue-specific elements including the 3-AF3 binding site, AT-rich element, Box II -like sequence, A-box, CAT-box, ARE, CCAAT-box, NON-box, circadian, MBSI, GCN4_motif, MSA-like, HD-Zip 1, HD-Zip 3, motif I, O2-site, and RY-element (S8 Data). AREs were found to be the most prevalent elements in the promoters of *GhFH* genes. Additionally, 11 phytohormone-related elements were identified, including ABREs, CGTCA motif-containing elements, AuxRR-core-containing elements, P-boxes, GARE motif-containing elements, SAREs, TCA elements, TATC-boxes, TGACG motif-containing elements, TGA-boxes, and TGA elements. These elements respond to abscisic acid (ABREs), auxin (AuxRR-core-containing elements, TGA motif-containing elements, and TGA-boxes), methyl jasmonate (MeJA) (CGTCA motif-containing elements and TGACG motif-containing elements), gibberellin (GARE motif-containing elements, TATC motif-containing elements, and P-boxes), and salicylic acid (SAREs and TCA elements). Moreover, 29 cis-regulatory elements were associated with light response, including C-boxes, Box 4 elements, G-boxes, and more. Notably, GT1 motif elements and G-boxes were particularly abundant among these light-responsive cis-regulatory elements, highlighting their significance in regulating gene expression under varying light conditions.

### 3.8. Analysis of *Ka/Ks* of *GhFH* genes

**Fig 7:**
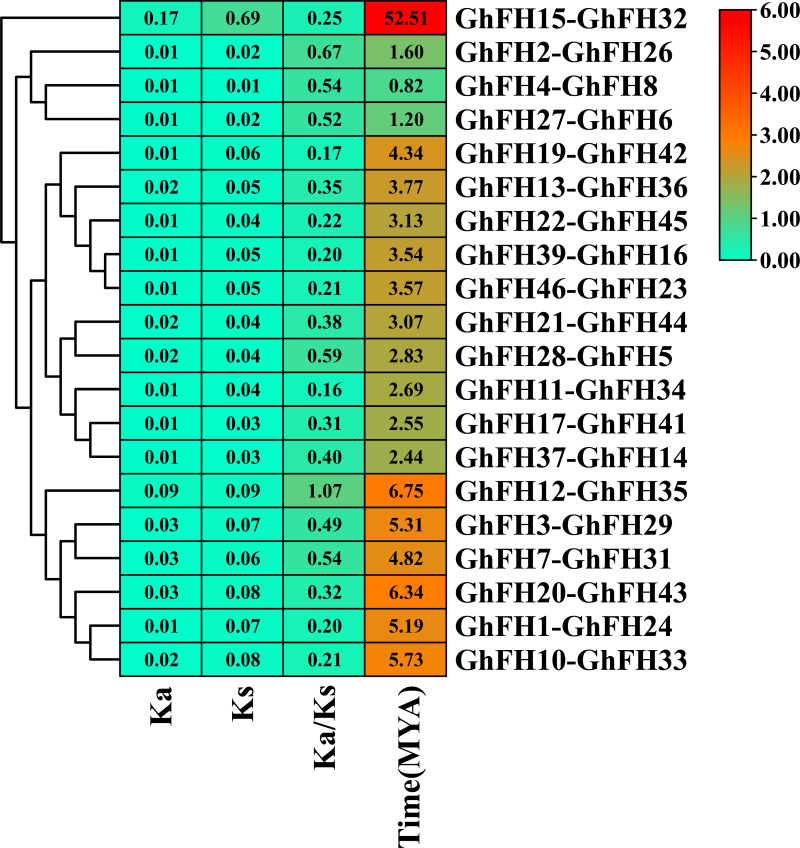
Estimation of gene duplication time for different gene pairs among *GhFH* genes, based on *Ks* and *Ka* values. Gene duplication analyses were conducted using TBtools software version-v1.116. *Ka* represents the number of nonsynonymous substitutions per site, while *Ks* represents the number of synonymous substitutions per site. The ratio of nonsynonymous (*Ka*) to synonymous (*Ks*) changes is represented by *Ka*/*Ks*.

The *Ka/Ks* analysis is a crucial indicator for understanding the evolution of gene duplication after separation from ancestors. A *Ka/Ks* value of 1 signifies neutral selection, while a value below 1 implies purifying selection and a value above 1 indicates positive or Darwinian selection [59]. The number of *Ka* and *Ks* along with their ratio *Ka/Ks*, were analyzed for 20 duplicated gene pairs. *Ks* values for these gene pairs varied from 0.01 (*GhFH4*-*GhFH8*) to 0.68 (*GhFH15*-*GhFH32*) with an average *Ks* of 0.08 (Fig 7). Among the 20 duplicated pairs, 19 exhibited a *Ka/Ks* ratio below 1, indicating purifying selection, except for one exceptional pair (*GhFH12*-*GhFH35*) with a ratio of 1.06 (S9 Data), suggesting positive selection. Additionally, *Ks* values were used to estimate the timing of gene duplication events (*GhFH* genes) in the evolutionary history of the cotton genome. Segmental and tandem duplication events in cotton were estimated to have occurred over a period ranging from 0.822 to 52.50 MYA, averaging 6.10 MYA.

### 3.9. Analysis of gene ontology (GO) of *GhFH* genes

**Fig 8:**
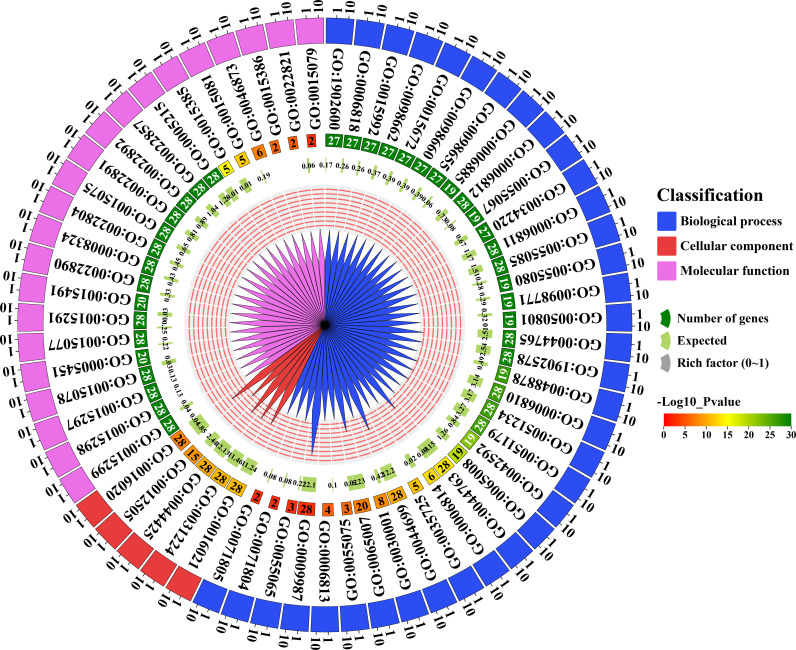
GO analysis of the FORMIN gene family in *G. hirsutum.* Circular heatmap for the predicted GO terms corresponding to the predicted *GhFH* genes presented for biological process, cellular components, and molecular function, whether the genes are associated or not. The *p*-value matching the GO terms is shown in the heatmap, using log10 (*p*-value).

GO analysis was performed to investigate the regulatory pathways and functions of the identified *GhFH* genes. A total of 59 unique GO IDs were identified each with their respective *p*-value. The identified GO terms were categorized into three groups: molecular functions (F), biological processes (P), and cellular components (C) (S10 Data). Among these, the biological processes group contained 48 GO terms (Fig 8). For instance, (GO: 0051258, *p*-value: 0.0000000074) coordinates the process of protein polymerization. Additionally, (GO:0030036, *p*-value: 0.000000014) controls the organization of the actin cytoskeleton, a necessary component for intracellular mobility and the maintenance of cell shape. Organelle organization is directed by (GO: 0006996, *p*-value: 0.000054) and the regulation of biological processes is controlled by (GO: 0050789, *p*-value: 0.0072). The cellular components and molecular functions categories exhibited 5 GO terms each including (GO: 0071944, *p*-value: 0.000029), (GO: 0009524, *p*-value: 0.000074), (GO: 0005886, *p*-value: 0.00029), (GO: 0005618, *p*-value: 0.0004), (GO: 0030312, *p*-value: 0.0004) for cellular components, and (GO: 0005515, *p*-value: 0.00000016), (GO: 0051015, *p*-value: 0.000014), (GO: 0003779, *p*-value: 0.0011), (GO: 0032403, *p*-value: 0.0033), (GO: 0008092, *p*-value: 0.0072), and (GO: 0044877, *p*-value: 0.0076) for molecular functions. Notably, (GO: 0030312, *p*-value: 0.0004) is associated with the external encapsulating structure while cytoskeletal protein binding is regulated by (GO: 0008092, *p*-value: 0.0072). Actin binding and actin filament binding are characterized by (GO: 0003779, *p*-value: 0.0011) and (GO: 0051015, *p*-value: 0.000014) respectively. Interestingly, 5 *GhFH* genes are linked to the cell periphery (GO:0071944, *p*-value: 0.000029) in the cellular components, and 12 *GhFH* genes can function as “protein binding (GO: 0005515, *p*-value: 0.00000016)” in the molecular functions category.

### 3.10. Collinearity analysis of *GhFH* genes

**Fig 9:**
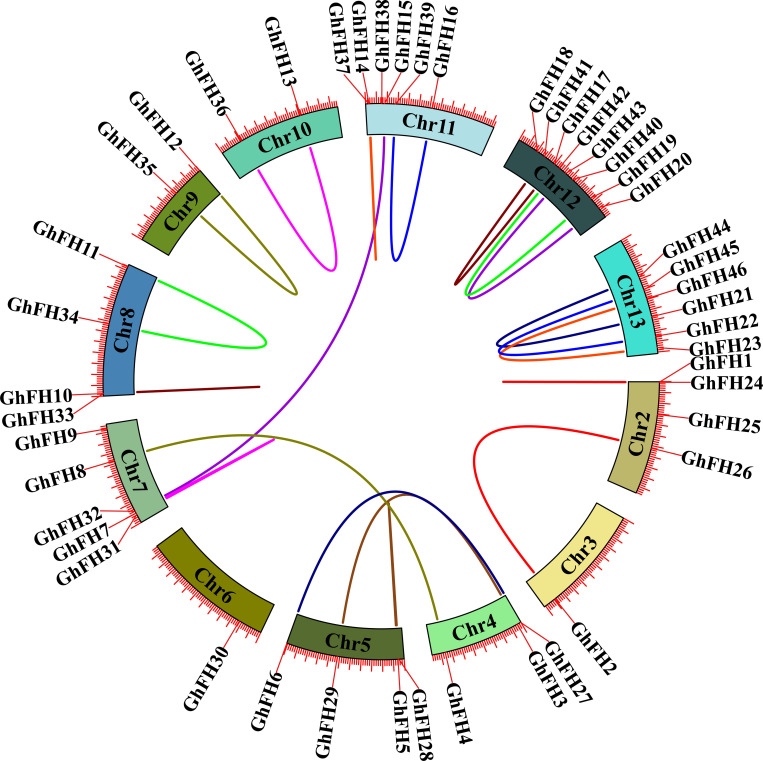
Collinearity analysis of the *GhFH* gene. Various colored rectangles represent chromosomes 2–13 within the *G. hirsutum* genome, while the colored lines linked between chromosomes represent segmental and tandem duplicated gene pairs.

The origins of the *GhFH* gene in *G. hirsutum* and their relationship with other homologous genes were determined through collinearity analysis. 46 *GhFH* genes were distributed unevenly across the 12 chromosomes. A total of 20 collinear pairs were identified among the 46 genes in *G. hirsutum* (Fig 9). Specifically, *GhFH2* was found to exhibit a collinear relationship with *GhFH26*, *GhFH16* with *GhFH27*, *GhFH3* with *GhFH29*, *GhFH7* with *GhFH38*, *GhFH4* with *GhFH8*, among. However, *GhFH30* located on chromosome 6, did not show any collinear pairing.

### 3.11. Analysis of synteny of *GhFH* genes with other plant species

**Fig 10:**
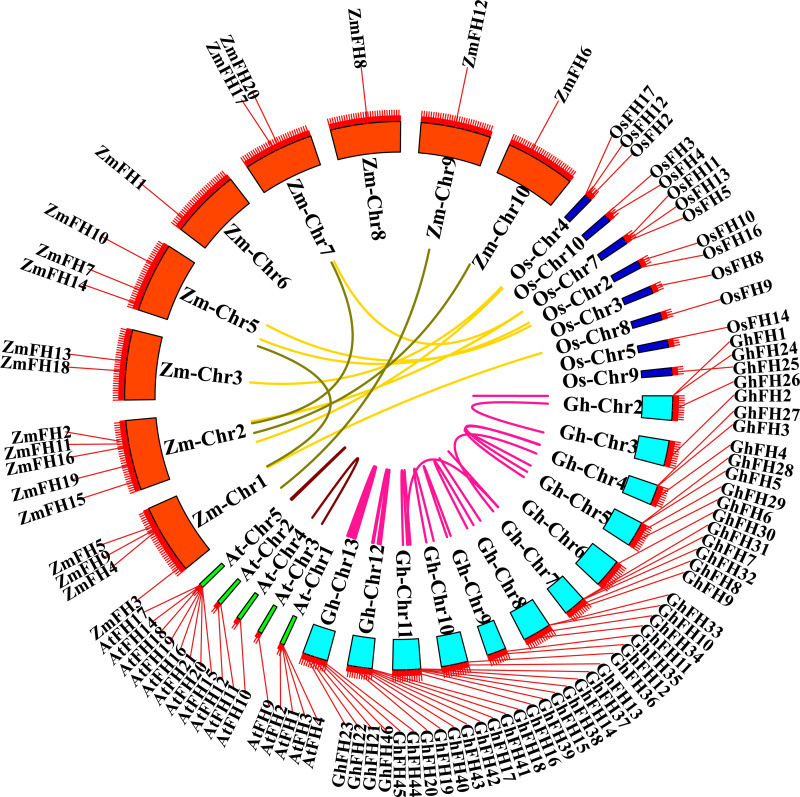
Synteny analysis of the FORMIN gene family among *G. hirsutum* and three other species, *A. thaliana, Z. mays, and* O. *sativa.* The yellow-colored lines represent the 7 syntenic gene pairs between *O. sativa* and *Z. mays*.

To explore the potential evolutionary connection between FH members found in different plant species, a synteny analysis was conducted between *G. hirsutum, A. thaliana, Z. mays, and* O. *sativa* (Fig 10). The analysis revealed that *G. hirsutum* lacked orthologous FH genes but displayed 20 pairs of paralogous genes. In contrast,7 orthologous FH gene pairs were identified between *Z. mays* and *O. sativa.* For instance, *ZmFH10* showed a syntenic relationship with *OsFH16*, *ZmFH18* with *OsFH12*, *ZmFH7* with *OsFH10*, *ZmFH11* with *OsFH11*, *ZmFH9* with *OsFH9*, *ZmFH20* with *OsFH13*, and *ZmFH15* with *OsFH2*, respectively. Additionally*, A. thaliana* exhibited only 2 paralogous FH gene pairs, highlighting unique evolutionary patterns within this species.

### 3.12. Analysis of transcription factor (TFs) and regulatory network of *GhFH*

TFs are the master regulators that control gene expression by binding to specific DNA sequences [60]. These TFs play crucial roles in how plants respond to various environmental challenges, including biotic and abiotic stresses, and regulate essential processes such as metabolism, growth, and development. Additionally, TFs coordinate plant defense mechanisms against a wide range of microbial pathogens [61–65]. In plants, various TF families exist, including ERF, MYB, bZIP, GATA, CBF/DREB1, SBP, G2-like, NAC, LBD, HSF, E2F/DP, C2H2, AP2/EREBP, TALE, WRKY, Dof, MIKC_MADS, BBR-BPC, TGA6, C2H2, BOS1 families, and others. These TFs serve as the master regulators, coordinating gene expression in response to environmental challenges, developmental cues, and internal signals [63,66,67].

**Fig 11:**
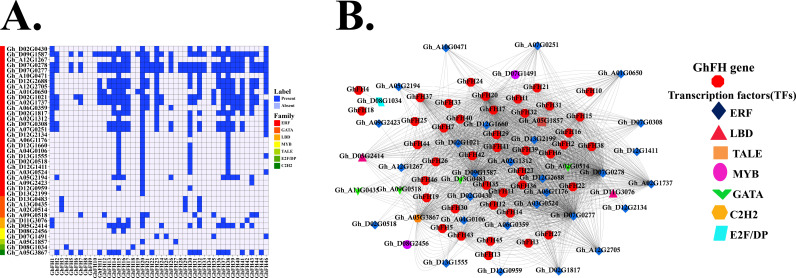
TFs analysis of *GhFH* genes. A. Distribution of TFs of *GhFH* genes represented by a heatmap. Dark blue color denotes the present of TFs and light blue denotes their absence. B. Regulatory network among the TFs and the predicted *GhFH* genes. Nodes are colored based on *GhFH* genes and TFs. Genes are represented in red color, and the TFs are represented by different colors. Different node symbols are used for different TF families.

In this study, among the 1581 TFs, 43 distinct TFs were identified to regulate *GhFH* genes. These TFs belong to 11 different families (ERF, C2H2, GATA, LBD, MYB, TALE, E2F/DP, BBR-BPC, G2-likeHSF, and SBP) Among these the ERF, GATA, MYB, and LBD families, comprising 36 TFs are believed to play a crucial role in the regulation of *GhFH* genes. Notably, these four TFs families contained 28, 4, 2, and 2 TFs, respectively, accounting for 83.72% of the 43 identified TFs. The interactions between the predicted *GhFH* genes and the major TF families (ERF, C2H2, GATA, LBD, MYB, TALE, E2F/DP) were also analyzed (Fig 11A and S11 Data).

The fundamental mechanisms of biological activities, including organ formation and homeostasis [68], stress response [69], plant defense [70], and signal transduction [71] rely on interaction networks. ERF family was found to be linked to all of the *GhFH* genes, except *GhFH4* and *GhFH18*. The C2H2 family was the second most prevalent, with a single TF (Gh_A05G3867) interacting with 20 *GhFH* genes. Additionally, 17 *GhFH* genes were associated with the LBD protein family and 12 *GhFH* genes were involved in interactions with the GATA family. The TALE family interacted with 3 *GhFH* genes, while the MYB TF family connected with 5 *GhFH* genes. Furthermore, the E2F/DP family was found to interact exclusively with *GhFH4* and *GhFH18 (*Fig 11B).

### 3.13. Analysis of protein-protein interaction (PPI)

**Fig 12:**
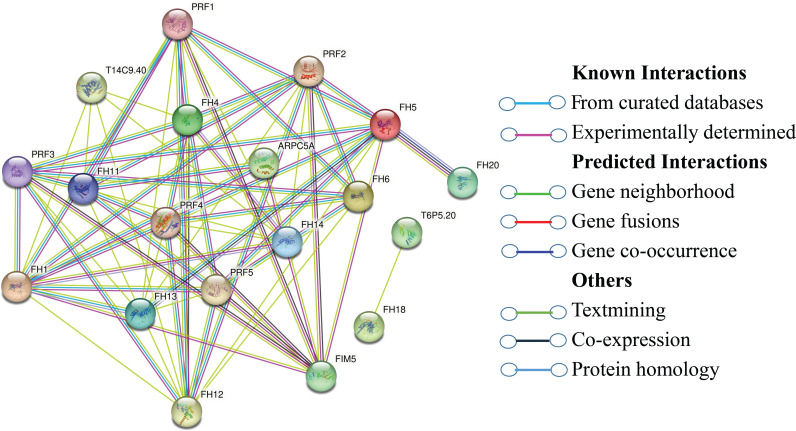
Protein-protein interactions between GhFH proteins and *Arabidopsis* proteins. The online STRING program was used to construct the network. Three-dimensional protein structures were shown at network nodes, and the colors of the lines indicate various data sources.

A PPI network analysis of GhFH proteins was conducted based on known *Arabidopsis* proteins. GhFH proteins that share significant similarities with *Arabidopsis* proteins were designated as STRING proteins. All 46 GhFH proteins exhibited interactions with known *Arabidopsis* proteins. Notably, GhFH25, GhFH24, GhFH15, GhFH32, GhFH38, GhFH1, GhFH36, and GhFH13 were identified as homologous to AtFH1, interacting with FH5, FH2, FIM5, ARPC5A, PRF1, PRF2, PRF3, PRF4, and PRF5 proteins. Similarly, GhFH40, GhFH21, and GhFH44, homologous to AtFH11, displayed strong interactions with FH12, ARPC5A, PRF1, PRF2, PRF3, PRF4, PRF5, and T14C9.40 proteins. GhFH30, GhFH23, and GhFH46, homologous to AtFH13, interacted with FH1, FH5, PRF1, PRF2, PRF3, PRF4, and PRF5 proteins (Fig 12). The pattern continued for other GhFH proteins, aligning with various AtFH proteins and forming distinct interaction groups. Additionally, GhFH43, GhFH14, GhFH31, GhFH37, GhFH20, GhFH18, GhFH7, GhFH9, GhFH8, GhFH35, GhFH4 and GhFH12 were homologous with AtFH20. GhFH29, GhFH42, GhFH33, GhFH19, GhFH22, GhFH3, GhFH10 and GhFH45 were homologous with AtFH5 and interact with FH20, FIM5, PRF1, PRF2, PRF3, PRF4, PRF5 proteins. Furthermore, GhFH41, GhFH16, GhFH39 and GhFH17 were homologous with AtFH6 and have a strong interaction between FH12, PRF1, PRF2, PRF3, PRF4, PRF5 proteins. GhFH6, GhFH26, GhFH2, GhFH27 were homologous with AtFH4.GhFH34, GhFH11 were homologous with AtFH14 and GhFH5, GhFH28 were homologous with AtFH18. AtFH18 has a strong interaction with T6P5.20. The results of the PPI analysis were consistent with the phylogenetic relationships, as evidenced by AtFH proteins within the same phylogenetic group as GhFH proteins (e.g., GhFH6, GhFH26, GhFH2, GhFH27 being homologous with AtFH4 and situated in the same phylogenetic group A, interact with each other).

### 3.14. Identification of microRNAs (miRNAs) targeting *GhFH* genes

**Fig 13:**
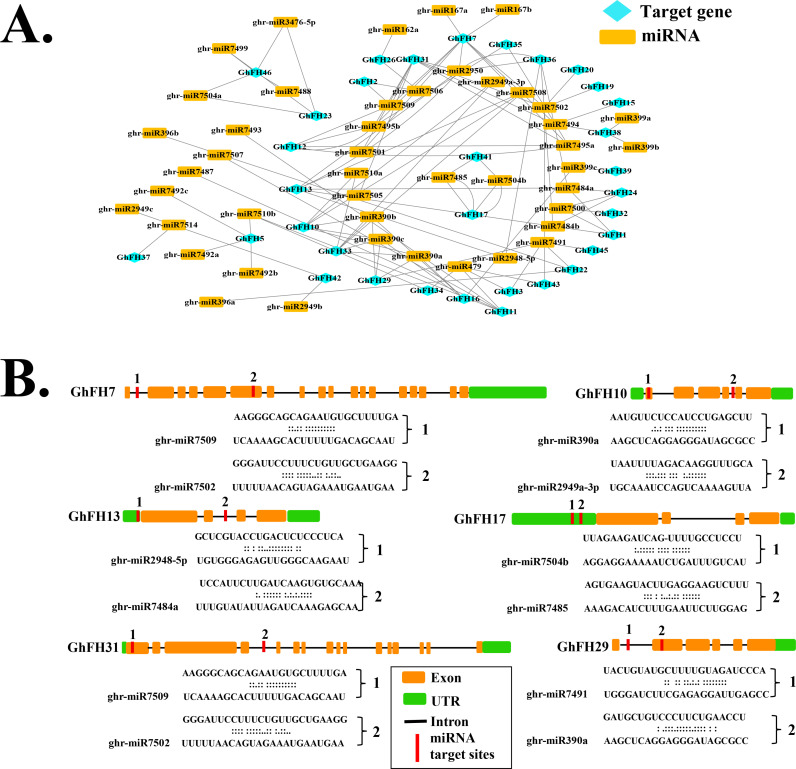
Predicted miRNAs targeting *GhFH* genes. A. The network diagram shows miRNAs predicted to target *GhFH* genes, with *GhFH* genes represented by rectangular shapes, and miRNAs by aqua ellipses. B. The schematic diagram displays *GhFH* genes targeted by miRNAs.

The role of miRNAs in gene regulation was investigated by identifying 45 putative ghr-miRNAs from 32 different families, targeting 33 *GhFH* genes. The regulatory mechanism of miRNAs in *GhFH* gene regulation was analyzed, with the findings illustrated through network diagrams (Fig 13A and B and S12 Data). Specific miRNA-family associations with *GhFH* genes were identified such as the ghr-miR390 family which targeted *GhFH10*, *GhFH16*, *GhFH29*, and *GhFH33*. Similarly, ghr-miR7484 targeted *GhFH36*, *GhFH24*, *GhFH13*, and *GhFH1*. The ghr-miR399 family influenced *GhFH16*, *GhFH38*, and *GhFH39*, while ghr-miR7495 targeted *GhFH7*, *GhFH35*, *GhFH31*, and *GhFH12*. Notably, the ghr-miR7502 family targeted five genes: *GhFH7*, *GhFH43*, *GhFH31*, *GhFH20*, and *GhFH19*. Other families such as ghr-miR7494, ghr-miR7504, and ghr-miR2950 showed similar patterns, targeting combinations of genes like *GhFH7*, *GhFH38*, *GhFH31*, *GhFH15* and *GhFH41*, *GhFH17*, *GhFH46*, *GhFH23*. Certain families targeted fewer genes for example, ghr-miR2949 and ghr-miR7492 each targeted *GhFH42* and *GhFH5*, respectively. Additionally, individual miRNAs like ghr-miR479, ghr-miR7491, and ghr-miR7508 play roles in regulating multiple genes, including GhFH43, GhFH34, GhFH3, GhFH11, and others. Unique miRNA-*GhFH* interactions were revealed, with miRNAs such as ghr-miR162a, ghr-miR7487, ghr-miR7488, ghr-miR7493, ghr-miR7505, ghr-miR7507, and ghr-miR7514 targeted genes such as GhFH26, GhFH11, GhFH46, GhFH31, and GhFH37. Notably, *GhFH7* was identified as the most targeted gene, with nine miRNAs from seven families regulating its expression. The ghr-miR7502 family was distinguished for targeting five genes: *GhFH7*, *GhFH43*, *GhFH31*, *GhFH20*, and *GhFH19.*

### 3.15. Analysis of *GhFH* gene expression in various tissues

**Fig 14:**
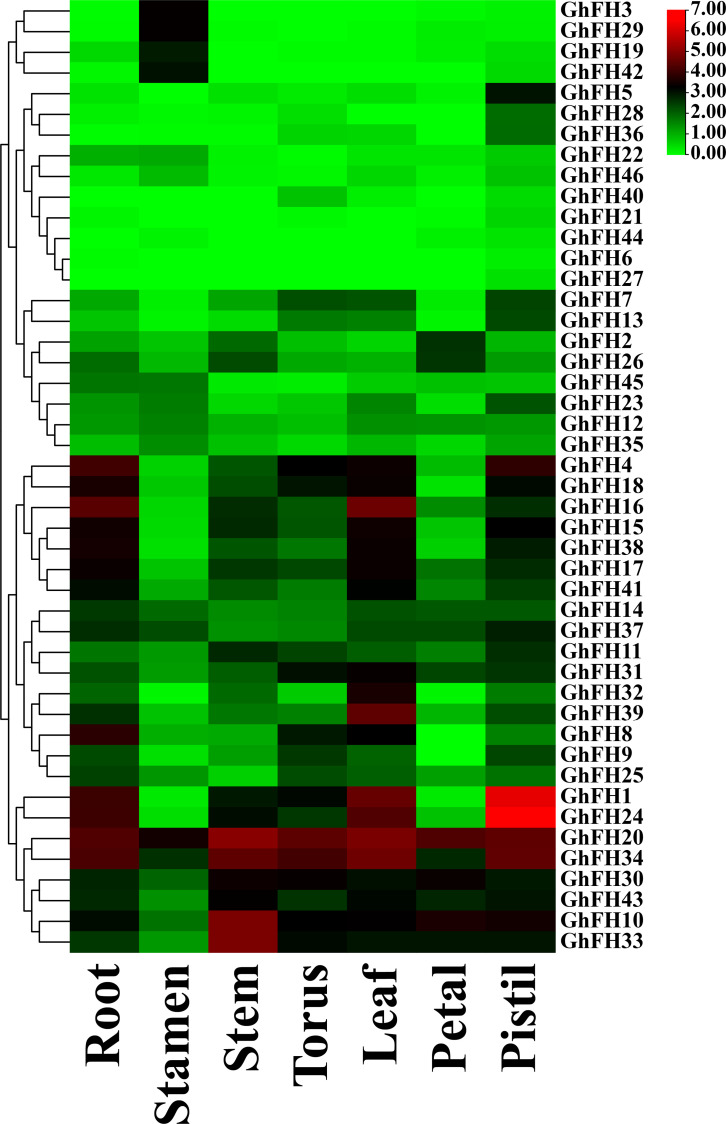
Tissue-specific expression of *GhFH* genes. A heatmap represents the expression profile of *GhFH* genes in root, stamen, stem, torus, leaf, petal, and pistil. *GhFH* gene names are listed on the right side of the heatmap, and tissue types are indicated at the bottom. Color intensity reflects the presence of protein signals corresponding to the genes.

The expression of all identified *GhFH* genes was examined across various tissues to explore their potential functions in the growth and development of *G. hirsutum* cultivar Texas Marker-1 (TM-1) based on RNA-seq data. The results indicated significant variation in the expression of *GhFH* genes in specific tissues such as the root, stamen, stem, torus, leaf, petal, and pistil. All 46 *GhFH* genes were expressed in the pistil, with the highest expression observed in this tissue. Expression levels were also high in the root (43 genes, 93.48%), in the stamen (41 genes, 89.13%) torus, (40 genes, 86.96%), leaf (39 genes, 84.78%), and stem and petal (38 genes, 82.61%). The *GhFH20* and *GhFH34* genes were highly expressed across all selected tissues compared to other *GhFH* genes with particularly high expression levels, of *GhFH24* and *GhFH1* in the pistil (91.74 FPKM and 73.44 FPKM, respectively) (Fig 14 and S13 Data).

### 3.16. Analysis of *GhFH* genes in response to different light conditions

**Fig 15:**
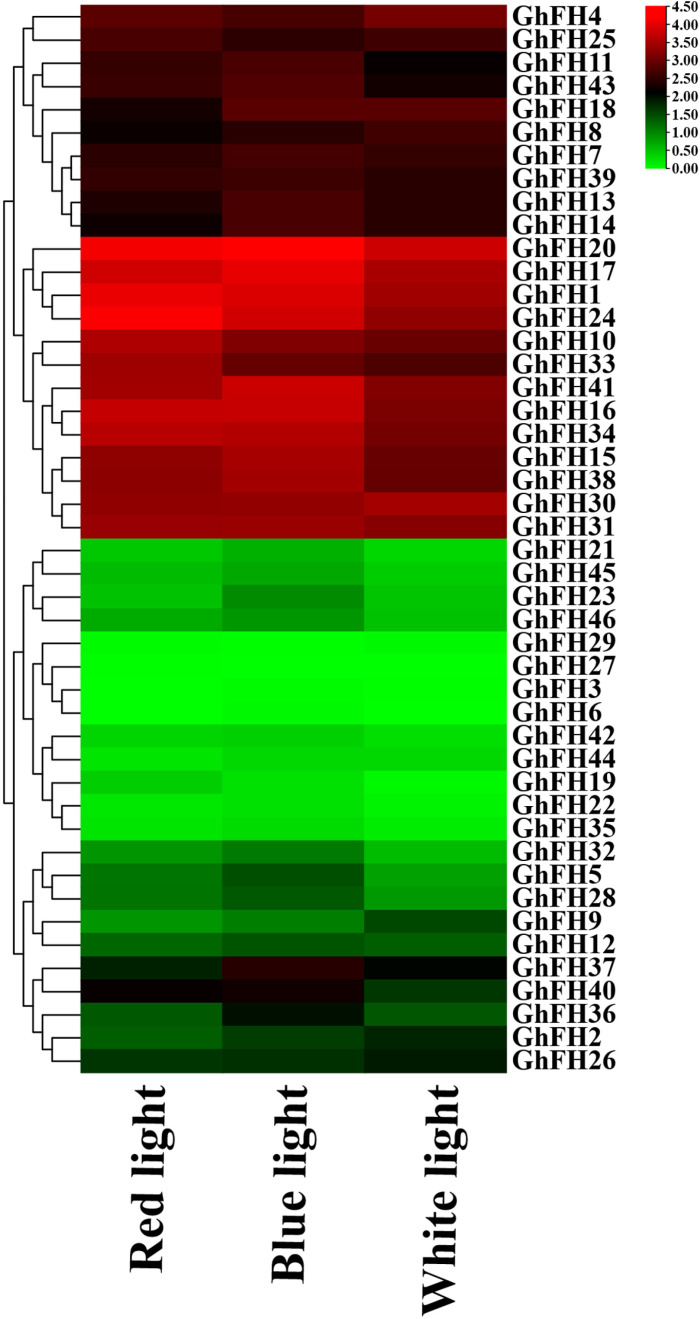
The expression pattern of *GhFH* under light conditions. *GhFH* gene names are listed on the right side of the heatmap and light treatments (red, blue, and white) are represented at the bottom of the heatmap. The color gradient from green to red indicates the expression levels.

The effect of light on the expression of the identified 46 *GhFH* genes was analyzed under three different light conditions: red, blue, and white. Among the 46 *GhFH* genes, 29 showed significantly higher expression under blue light compared to red and white light, while only 8 genes exhibited enhanced expression under white light (Fig 15 and S14 Data). *GhFH20* had the highest expression, level under blue light, reaching 17.86 FPKM. *GhFH27* was not expressed under blue along with white lights, and *GhFH6* was not expressed under red and white lights. However, *GhFH1*, *GhFH10*, *GhFH20*, *GhFH24*, and *GhFH30* displayed a good level of expression under all three light conditions.

### 3.17. Analysis of *GhFH* genes in response to abiotic stress conditions

**Fig 16:**
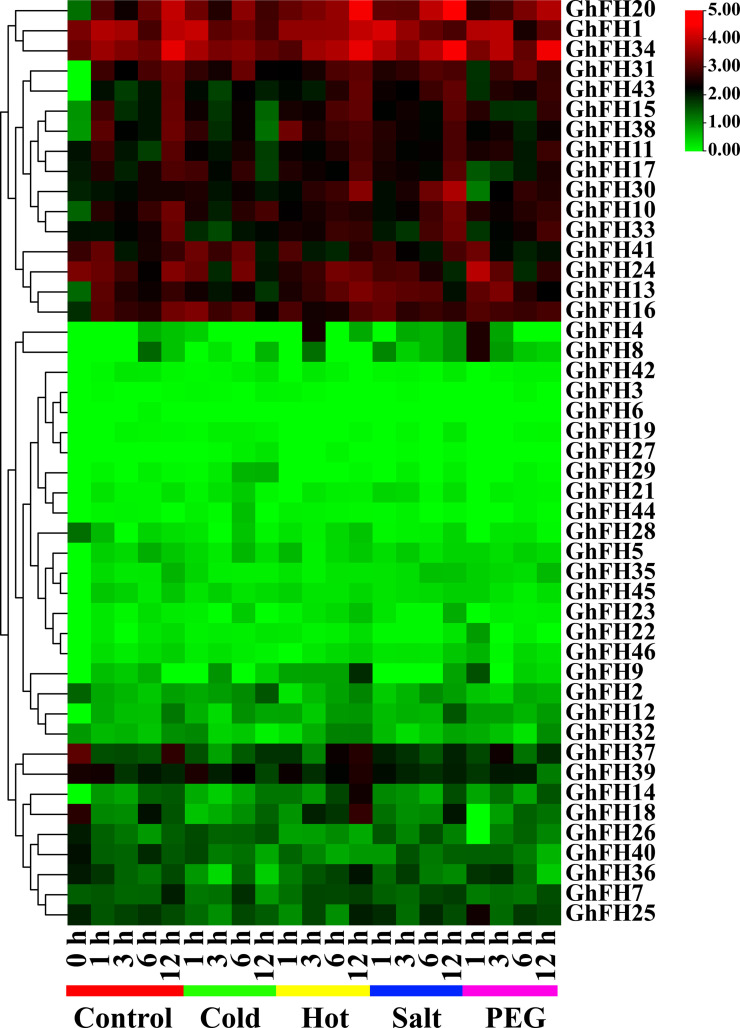
Expression profiles of *GhFH* genes in leaf tissue under different abiotic stress conditions. The FPKM values were converted to the Log2 format and compared to the control. Expression data were clustered and displayed using TBtools version-v1.116 with a color gradient indicating expression levels from low to high (ranging from green to red) depicted on the right side of the heatmap.

The response of identified *GhFH* genes in *G. hirsutum* to abiotic stresses (cold, hot, salt, and PEG) was analyzed using RNA-Seq data. Gene expression was studied in leaf tissue at different time points (1h, 3h, 6h, and 12h) following exposure to these stresses. The findings revealed varied expression patterns, with some genes being up-regulated, and others down-regulated in response to stresses (Fig 16 and S15 Data). Based on the differential expression patterns, the *GhFH* genes were grouped into three categories: a) some *GhFH*s had very low expression levels, b) some had low to medium expression levels, and c) some had high expression levels throughout treatments. The results indicated that *GhFH9*, *GhFH20*, and *GhFH30* exhibited higher expression in hot environments compared to controls. *GhFH20* and *GhFH30* genes also showed increased expression when exposed to salt. The expression of the *GhFH34* gene gradually increased under hot and salt treatment. *GhFH17*, *GhFH20*, and *GhFH33* were up-regulated during PEG treatment over treatment time. In contrast, all 46 identified *GhFH* genes showed decreased activity in leaf tissue under cold stress. *GhFH6* didn’t express under any of the abiotic stresses(cold, hot, salt, and PEG).

## 4. Discussion

Cotton cultivation is recognized as the backbone of the economic prosperity in numerous nations. The textile industry primarily relies on cotton as its main source of natural fiber source [72]. However, the production of this valuable crop is challenged by various abiotic stresses including salt, drought, cold, and heavy metal toxicity. These abiotic stress conditions significantly limit plant distribution, alter growth and development, and reduce crop productivity [73,74]. FORMIN proteins, which are crucial for cell growth and development, are ubiquitous in plants [17,75]. However, their involvement in mediating morphological changes in response to various environmental cues remains unclear [76]. FORMIN proteins have been successfully identified in *Arabidopsis* and rice, as well as in several angiosperms, including tobacco, sorghum, tomato, pea, wheat, and soybean [77].

In this study, *in silico* characterization of the identified FH genes in *G. hirsutum* was performed. A total of 46 FH genes were retrieved from *G. hirsutum*, distributed unevenly across the 12 chromosomes. It has been suggested that genes within the same family may be distributed on different chromosomes due to their involvement in various functions [78]. Proteins are classified as hydrophobic or hydrophilic based on their GRAVY value, with a positive value indicating hydrophobicity and a negative value implying hydrophilicity [79]. Based on the physio-chemical properties, all identified GhFH proteins except for GhFH37, with a value of 1.08, exhibited an average GRAVY value of less than 1, indicating that all proteins were hydrophobic (non-polar).

To further understand the evolutionary relationship among the 46 GhFH proteins a phylogenetic tree was constructed, including, 21 proteins from AtFH, 19 proteins from MtFH, 17 proteins from OsFH, and 20 proteins from ZmFH. These findings suggest that genes in *G. hirsutum*, *O. sativa*, and *Z. mays* remained highly conserved throughout evolution. The placement of exons and introns, essential for the evolution of gene families [80] was also analyzed. Genes carry the information required for reproduction and survival [81] and the analysis revealed a comparatively high level of structural variation among the *GhFH* genes. Genes clustered together within the phylogenetic tree shared significant similarities in their exon-intron structure [54]. The results of conserved domain and motif analysis showed that GhFH proteins within the same group, according to the phylogenetic tree, had similar motif distribution patterns and domain compositions. Besides the FH2 domain which was present in all GhFH proteins, certain individual domains were exclusively present in specific groups.

The subcellular locations of specific proteins play a crucial role in the biological processes and activities of plants [82,83]. FORMIN proteins have two key domains: the profilin-rich FH1 domain, which is essential for elongating actin filaments, and the FH2 domain, which is crucial for nucleating actin filaments [84]. Accordingly, our findings indicate that GhFH proteins are predominantly located in chloroplasts and the nucleus, suggesting a possible function as nucleation factors for actin filament nucleation. Proteins in chloroplasts are also involved in carbon fixation, amino acid biosynthesis, photosynthesis, and redox homeostasis. Chilling stress signals are also recognized by chloroplasts through membranes and photoreceptors, and their homeostasis is maintained and photosynthesis is promoted by regulating the state of lipid membranes [85]. The plant nucleus, which regulates gene expression is crucial for plants to adapt to abiotic stresses like drought, salinity, and extreme temperatures [74]. GhFH proteins present in the nucleus likely participate in the regulation of gene expression, signal transduction, sensing, or other essential nuclear processes crucial for cellular function. Additionally, 60.84% of GhFH proteins inhabit mitochondria suggesting a potential correlation with cellular respiration and energy metabolism.

CAREs are essential for coordinating cellular responses to environmental stimuli and developmental cues by regulating the TFs [86]. In this study, 62 types of CAREs (light-responsive, tissue-specific, stress-responsive, and phytohormone-responsive) were confirmed in the promoters of *GhFHs*. The most frequently observed light-responsive motifs in cotton *GhFH* genes were Box 4, GT1-motif, G-Box, GATA-motif, and TCT-motif. Photosynthesis, a crucial physiological process related to light response, is typically observed in plant leaves. Early flowering, which can lead to high productivity, may be caused by a high photosynthesis rate [87]. Plant hormones or growth regulators (PGRs) play a vital role in plant seed germination, growth, development, and metabolic activities [88,89]. In this study, several important hormone-responsive CAREs were identified, including ABRE which is involved in abscisic acid responsiveness [90] and controls the expression of genes that respond to salt and dehydration in rice and *Arabidopsis* [91]. Other elements identified include the, GC motif and CGTCA-motif associated with anoxic-specific inducibility [92], AuxRR-core related to auxin responsiveness [88], TCA-element related to salicylic acid responsiveness [93] were identified. Additionally, LTR (involved in low-temperature response), TC-rich repeats (engaged in stress response and defense) and MBS (associated with drought inducibility) [91,94,95] were also found. These findings suggest that *GhFH* genes have a significant influence on responses to abiotic stress, phytohormone reactions, and defense-related signal transduction. The number of (*Ka*) and (*Ks*), along with their ratio, serves as a crucial tool for identifying proteins under selective pressure [96]. The calculated Ka/Ks ratio of all *GhFH* gene pairs was found to be less than 1 except for one exceptional pair (*GhFH12-GhFH35*) which had a ratio of 1.06. This analysis suggests that these genes may have undergone limited functional divergence and experienced strong purifying selection pressure during their evolutionary history. Gene duplication, including tandem and segmental duplication is regarded as a primary driving force in the evolution of genetic systems and genomes. It also enables organisms to adapt to their changing environments [97,98]. In this study, out of 46 *GhFH* genes identified in the *G. hirsutum* genome, 30 tandem duplications (65.28%) and 10 segmental duplications were observed. This detailed pattern highlights the role that tandem and segmental duplication events played in the development and expansion of the *GhFH* gene family.

To explore the functions of the identified *GhFH* genes, GO analysis was performed. A total of 59 unique GO IDs were identified, covering biological processes, molecular functions, and cellular components. Among these, biological processes exhibited the highest diversity with 48 essential terms, highlighting their crucial role in various biological functions. Distinct *p*-values were observed for each GO term, with GO:0044877 showing the highest number of *p*-value: 0.0076 and GO: 0030838 showing the lowest number of *p*-value: 0.00000000041, providing valuable insights into the significance of these terms in biological systems. Further investigation is needed to fully understand the functional significance of these findings.

Based on the results of collinearity and synteny analysis, it is predicted that the *GhFH* gene in *G. hirsutum* may have undergone duplication events during evolution, resulting in multiple copies of the gene in the genome. It was also demonstrated that the collinear gene pairs of *GhFH* genes have been maintained throughout cotton evolution, except *GhFH30*, located on chromosome 6, which did not exhibit such collinear gene pairing. Comparative synteny mapping revealed no syntenic pair of *GhFH* genes with other species *(Arabidopsis*, rice, and maize). Notably, the synteny analysis revealed the presence of 7 syntenic FH gene pairs between *Z. mays* and *O. sativa*. The presence of these syntenic gene pairs underscores the potential functional significance and evolutionary conservation of FH genes in plants, indicating possible functional similarities or shared evolutionary history among these FH genes.

TFs are bound to specific CARE regions in the promoters of target genes to regulate gene expression. Crucial regulators of numerous biological processes include ERF, C2H2, GATA, LBD, MYB, TALE, E2F/DP, and other plant TFs [99–101]. The Ethylene Response Factor (ERF) is essential for the response pathway in plants and for ethylene (ET) signaling. The ability of plants to endure challenging environments for extended periods is enhanced by the response of ERF to multiple plant hormones. PGRs such as abscisic acid (ABA) and ET can stimulate ABA-ET-dependent or independent stress-responsive (SR) genes through the action of certain AP2/ERF families [102]. The adaptability of tomatoes to salt and drought is improved by ERF, specifically Slerf5 (ERF5) [103]. Additionally, overexpression of Tsrf1, an ERF TF, has been shown to increase drought tolerance in rice [104]. MYB TFs are found in large quantities in plant systems; constituting approximately 9% of the entire TF family in *A. thaliana* [105]. MYB TFs influence numerous biological processes, including plant growth and development, cell shape and pattern creation, metabolism of physiological activities, and responses to biotic and abiotic stressors [106]. GATA TFs, a family of DNA-binding proteins found in many plant species, are connected to the regulation of transcription in plants that rely on light and nitrate [107]. The adaptability of GATA TFs is demonstrated by their interactions with biotic and abiotic stressors. Expression profiles show that GATA genes in rice, *Brassica juncea*, C*ucumis sativus*, and pepper respond to various abiotic stressors, including high temperatures, salinity, cold, and drought [108–111].

LBD TFs play pivotal roles in regulating the growth and development of various plant species. They are actively involved in secondary growth promotion, root, stem, leaf, and corolla growth, as well as the initiation and regulation of metabolic activities. Additionally, LBD genes contribute to the differentiation between terminal meristem primordia and lateral organ primordia. In higher plants, LBD genes have a significant influence on the development and maturation of both aerial and root. They are also essential for the metabolism of nitrogen and anthocyanins [112–114]. The C2H2 TFs family encodes proteins that are crucial for plant development, growth, and resistance to biological stress [115].

Within the TFs superfamily GARP (Golden2, ARR-B, and Psr1) domain, the G2-like proteins are unique members [116]. These G2-like TFs are critical for the development and maturation of chloroplasts [117–119] and have been associated with various defense mechanisms in various organisms, including response to biotic and abiotic stress [120–122]. Regulatory network analysis predicts a broad range of expression patterns for the *GhFH* genes and TFs in cotton. The results indicate that all genes interact with the ERF family except for two genes, providing strong evidence that these *GhFH* genes are associated with various plant hormones, which help plants survive in stressful environments for longer periods.

PPI network analysis reveals the activities of specific gene families associated with known proteins [123]. The study showed that 46 GhFH proteins share homology and establish strong interactions with 10 known *Arabidopsis* proteins, including AtFH1, AtFH11, AtFH13, AtFH14, AtFH18, AtFH20, AtFH4, AtFH5, and AtFH6. These FH proteins play crucial roles in *Arabidopsis*. For example, the AtFH5-GFP fusion protein, essential for cell division, accumulates in the cell plate. Additionally, AtFH6 regulates polarized growth by adjusting the assembly of actin cables [17]. Other reports indicate that AtFH8 influences root and root hair development by modifying the distribution of the actin cytoskeleton [18,124]. AtFH14 interacts with microtubules and microfilaments to regulate cell division [125]. The results suggest that GhFH family proteins may have similar functions. The study identified 45 putative ghr-miRNAs from 32 different families. Among these ghr-mir390a/b/c, ghr-miR2950, ghr-miR7491, ghr-miR7484a/b, and ghr-miR7502 were found to target most of the *GhFH* genes (Table 2).

**Table 2:** Information about abundant miRNA ID, functions, and their targeted *GhFH* genes.

miRNA ID	Functions	Targeted genes	References
ghr-mir390a/b/c	Responds to salt stress	GhFH10, GhFH16, GhFH29, GhFH33	[126]
ghr-miR2950	Role in the growth of cotton fiber	GhFH7, GhH13, GhFH31, GhFH36	[127]
ghr-miR7491	Involves in stress reactions, pest management, and fertility restoration	GhFH3, GhFH22, GhFH29, GhFH45	[128]
ghr-miR7484a/b	Regulates cotton fiber development	GhFH1, GhFH13, GhFH24, GhFH36	[127]
ghr-miR7502	Role in response to salt stress	GhFH7, GhFH19, GhFH20, GhFH31, GhFH43	[129]

These results suggested that the discovered gh-miRNAs may play important roles in overcoming various stresses by altering the transcriptional levels of *GhFH* genes in *G. hirsutum*, although further wet lab experiment is needed to confirm this theory.

Gene expression profiling provides important insights into determining gene functions [130]. In the current study, diverse expression levels of *GhFH* genes were observed among the selected tissues. All genes were expressed in the pistil, which is the female reproductive part of a flower, and responsible for receiving pollen and producing seeds [131]. This indicates that our identified genes play an important role in reproduction.

As the climate changes progress and arable land decreases, it is crucial to investigate how environmental stress affects the growth of important crops. Light serving as an essential energy source and a developmental cue for plants, can also induce stress and influence how plants respond to various stress factors [132]. Analysis of expression profiles from previous transcriptome data under red, blue, and white light conditions revealed that *GhFH* genes were highly expressed across all three conditions, strongly complementing the CARE analysis data. Specifically, *GhFH1*, *GhFH10*, *GhFH20*, *GhFH24*, and *GhFH30* exhibited the highest level of expression across the three light conditions. The tissue-specific expression also showed that 39 out of 46 *GhFH* genes had relatively high expression levels in the leaf, and almost all of those FORMIN genes demonstrated elevated expression levels under hot treatments. *GhFH9*, *GhFH20*, and *GhFH30* genes had greater expression profiles in hot conditions compared to controls. High expression of *GhFH17*, *GhFH20*, *GhFH30*, *GhFH33,* and *GhFH34* genes were high under salt and PEG treatments. Polyethylene glycol (PEG) an osmotic priming agent, helps to reduce the damage from abiotic stresses [133]. In conclusion, these findings indicate that these *GhFH* genes may play important roles in how plants respond to stress like heat, salt, and PEG and these patterns may be further explored in subsequent research.

## 5. Conclusions

In this study, the FORMIN gene family in *G. hirsutum* was systematically and scientifically identified and characterized. A total of 46 *GhFH*s were identified, distributed across 12 chromosomes. According to the phylogenetic tree, GhFHs were categorized into five groups and were found to be closely related to OsFHs and MtFHs. The gene structure analysis of the 46 *GhFH* genes revealed the structural diversity of genes in *G. hirsutum*. CAREs and GO analysis elucidated the functions of FORMINs in *G. hirsutum*, particularly in plant development and stress-related activities. *Ka*/*Ks* analysis revealed the evolutionary history of the *GhFH* genes (0.822- 52.50) MYA. Collinearity analysis showed that gene duplication events facilitated the expansion of the FORMIN family in *G. hirsutum.* The majority of *GhFH*s have ERF TFs that respond to various environmental stresses. Two genes, *GhFH20* and *GhFH30*, showed increased expression when exposed to heat and salt stresses. Additionally, the expression of GhFH34 gradually increased with hot and salt treatments. These genes were also highly expressed under light conditions. These findings provide a foundation for understanding the roles of *GhFH* genes and manipulating *GhFH* gene expression in *G. hirsutum* could potentially lead to the development of cotton varieties that are more resilient to environmental stresses.

## Supporting information

S1 Data Peptide sequences of *GhFH* gene family. (TXT)

S2 Data Peptide sequences of *AtFH*, *OsFH*, *MtFH*, *ZmFh,* and candidate *GhFH* gene families were used for the construction of a phylogenetic tree. (TXT)

S3 Data Genomic sequences of *GhFH* gene family. (TXT)

S4 Data CDS of *GhFH* gene family. (TXT)

S5 Data The promoter region of *GhFH* gene family. (TXT)

S6 Data Distribution of *GhFH* genes. (DOCX)

S7 Data Number of introns and exons in *GhFH* genes. (DOCX)

S8 data The predicted *cis*-acting regulatory elements of the upstream promoter region (2.0 kb genomic sequences) of *GhFH* gene family member. (XLSX)

S9 Data Time of gene duplication estimated for different paralogous pairs of *GhFH* genes based on Ka and Ks values. (XLSX)

S10 Data The details GO analysis of the predicted *GhFH* genes was performed using the Plant Transcription Factor Database (Plant TFDB, http://planttfdb.cbi.pku.edu.cn/). (XLSX)

S11 Data Identified the main 7 TF families associated with the regulation of identified *GhFH* genes. (XLSX)

S12 Data miRNA prediction of targeted GhFHs. The miRNA data was downloaded from psRNATarget Server18. (DOCX)

S13 Data Tissue-specific expression profiles of *GhFH* genes retrieved from NCBI (accession number SRA: PRJNA248163). (XLSX)

S14 Data Expression profiles of *GhFH* genes under light conditions (accession number SRA: PRJNA765172). (XLSX)

S15 Data Expression profiles of *GhFH* genes under abiotic stress like cold, heat, salt, and PEG (accession number SRA: PRJNA248163). (XLSX)

S1 Fig The sequence logos of the 20 motifs found in the GhFH proteins. (TIF)
