# Supplementary material for: Genome-wide identification and characterization of FORMIN gene family in cotton (Gossypium hirsutum L.) and their expression profiles in response to multiple abiotic stress treatments
Source: PLoS One. 2025 Mar 3;20(3):e0319176. doi: 10.1371/journal.pone.0319176 (PMC11875364; doi:10.1371/journal.pone.0319176)
Supplement: S6 Data — (DOCX) [file pone.0319176.s006.docx]

**S6 Data.** *GhFH* gene family members distribution among groups based on phylogenetic analysis with *A. thaliana*, M. *truncatula*, *O. sativa*, and Z. *mays*.

| **Group** | **Number of *GhFH* genes** | **Gene ID** |
| --- | --- | --- |
| A | 4 | GhFH2, GhFH6, GhFH26, GhFH27 |
| B | 19 | GhFH4, GhFH5, GhFH7, GhFH8, GhFH9, GhFH11, GhFH12, GhFH14, GhFH18, GhFH20, GhFH23, GhFH28, GhFH30, GhFH31, GhFH34, GhFH35, GhFH37, GhFH43, GhFH46 |
| C | 8 | GhFH3, GhFH10, GhFH19, GhFH22, GhFH29, GhFH33, GhFH42, GhFH45 |
| D | 3 | GhFH21, GhFH40, GhFH44 |
| E | 12 | GhFH1, GhFH13, GhFH15, GhFH16, GhFH17, GhFH24, GhFH25, GhFH32, GhFH36, GhFH38, GhFH39, GhFH41 |
