# Supplementary material for: Genome-wide identification and characterization of FORMIN gene family in cotton (Gossypium hirsutum L.) and their expression profiles in response to multiple abiotic stress treatments
Source: PLoS One. 2025 Mar 3;20(3):e0319176. doi: 10.1371/journal.pone.0319176 (PMC11875364; doi:10.1371/journal.pone.0319176)
Supplement: S7 Data — (DOCX) [file pone.0319176.s007.docx]

**S7 Data.** *In silico* predicted the number of introns and exons in *GhFH* genes.

| **Group** | **Gene name** | **Intron** | **Exon** |
| --- | --- | --- | --- |
| **A** | GhFH2 | 1 | 2 |
|  | GhFH6 | 1 | 2 |
|  | GhFH26 | 1 | 2 |
|  | GhFH27 | 1 | 2 |
| **B** | GhFH4 | 3 | 4 |
|  | GhFH5 | 16 | 17 |
|  | GhFH7 | 17 | 18 |
|  | GhFH8 | 3 | 4 |
|  | GhFH9 | 3 | 4 |
|  | GhFH11 | 17 | 18 |
|  | GhFH12 | 16 | 17 |
|  | GhFH14 | 13 | 14 |
|  | GhFH18 | 3 | 4 |
|  | GhFH20 | 16 | 17 |
|  | GhFH23 | 16 | 17 |
|  | GhFH28 | 13 | 14 |
|  | GhFH30 | 16 | 17 |
|  | GhFH31 | 15 | 16 |
|  | GhFH34 | 13 | 14 |
|  | GhFH35 | 13 | 14 |
|  | GhFH37 | 13 | 14 |
|  | , GhFH43 | 16 | 17 |
|  | GhFH46 | 15 | 15 |
| **C** | GhFH3 | 6 | 7 |
|  | GhFH10 | 5 | 6 |
|  | GhFH19 | 5 | 6 |
|  | GhFH22 | 5 | 6 |
|  | GhFH29 | 5 | 6 |
|  | GhFH33 | 5 | 6 |
|  | GhFH42 | 5 | 6 |
|  | GhFH45 | 5 | 6 |
| **D** | GhFH21 | 3 | 4 |
|  | GhFH40 | 3 | 4 |
|  | GhFH44 | 3 | 4 |
| **E** | GhFH1 | 3 | 4 |
|  | GhFH13 | 3 | 4 |
|  | GhFH15 | 3 | 4 |
|  | GhFH16 | 3 | 4 |
|  | GhFH17 | 3 | 4 |
|  | GhFH24, | 3 | 4 |
|  | GhFH25 | 1 | 2 |
|  | GhFH32 | 3 | 4 |
|  | GhFH36 | 3 | 4 |
|  | GhFH38 | 3 | 4 |
|  | GhFH39 | 3 | 4 |
|  | GhFH41 | 3 | 4 |
