# Supplementary material for: Genome-wide identification and characterization of FORMIN gene family in cotton (Gossypium hirsutum L.) and their expression profiles in response to multiple abiotic stress treatments
Source: PLoS One. 2025 Mar 3;20(3):e0319176. doi: 10.1371/journal.pone.0319176 (PMC11875364; doi:10.1371/journal.pone.0319176)
Supplement: S12 Data — (DOCX) [file pone.0319176.s012.docx]

**S12 Data.** miRNA prediction of targeted *GhFH*s. The miRNA data was downloaded from psRNATarget Server18(<https://www.zhaolab.org/psRNATarget/analysis?function=2>)**.**

| **miRNA ID** | **Target ID** | **miRNA length** | **Target start** | **Target end** | **miRNA aligned fragment** |
| --- | --- | --- | --- | --- | --- |
| ghr-miR390a | GhFH10 | 21 | 328 | 348 | AAGCUCAGGAGGGAUAGCGCC |
| ghr-miR390b | GhFH10 | 21 | 328 | 348 | AAGCUCAGGAGGGAUAGCGCC |
| ghr-miR390c | GhFH10 | 21 | 328 | 348 | AAGCUCAGGAGGGAUAGCGCC |
| ghr-miR7507 | GhFH11 | 24 | 529 | 552 | AAGGUAGUGAAGUAGGCAAUUGGG |
| ghr-miR2950 | GhFH7 | 21 | 2055 | 2075 | UGGUGUGCAGGGGGUGGAAUA |
| ghr-miR7492a | GhFH5 | 23 | 877 | 899 | CUAUAGAACAUGAUCUUUAGCGG |
| ghr-miR7492b | GhFH5 | 23 | 877 | 899 | CUAUAGAACAUGAUCUUUAGCGG |
| ghr-miR7492c | GhFH5 | 23 | 877 | 899 | CUAUAGAACAUGAUCUUUAGCGG |
| ghr-miR7501 | GhFH31 | 24 | 2757 | 2780 | AUAUCUGAUUCUGACACGAAAAAA |
| ghr-miR7506 | GhFH26 | 22 | 1 | 22 | AUGUCUGGGACAUGGCGUUGGC |
| ghr-miR7506 | GhFH2 | 22 | 1 | 22 | AUGUCUGGGACAUGGCGUUGGC |
| ghr-miR7508 | GhFH13 | 21 | 607 | 627 | CAAGAAAAGAAGUCGGGAGAG |
| ghr-miR7510a | GhFH33 | 24 | 397 | 420 | AAGGUCAUGAUCUUUAGCGGCGUU |
| ghr-miR7510a | GhFH10 | 24 | 397 | 420 | AAGGUCAUGAUCUUUAGCGGCGUU |
| ghr-miR2948-5p | GhFH36 | 22 | 333 | 354 | UGUGGGAGAGUUGGGCAAGAAU |
| ghr-miR2948-5p | GhFH13 | 22 | 318 | 339 | UGUGGGAGAGUUGGGCAAGAAU |
| ghr-miR2950 | GhFH13 | 21 | 558 | 578 | UGGUGUGCAGGGGGUGGAAUA |
| ghr-miR390a | GhFH33 | 21 | 328 | 348 | AAGCUCAGGAGGGAUAGCGCC |
| ghr-miR390a | GhFH29 | 21 | 1228 | 1248 | AAGCUCAGGAGGGAUAGCGCC |
| ghr-miR390a | GhFH16 | 21 | 676 | 696 | AAGCUCAGGAGGGAUAGCGCC |
| ghr-miR390b | GhFH33 | 21 | 328 | 348 | AAGCUCAGGAGGGAUAGCGCC |
| ghr-miR390b | GhFH29 | 21 | 1228 | 1248 | AAGCUCAGGAGGGAUAGCGCC |
| ghr-miR390b | GhFH16 | 21 | 676 | 696 | AAGCUCAGGAGGGAUAGCGCC |
| ghr-miR390c | GhFH33 | 21 | 328 | 348 | AAGCUCAGGAGGGAUAGCGCC |
| ghr-miR390c | GhFH29 | 21 | 1228 | 1248 | AAGCUCAGGAGGGAUAGCGCC |
| ghr-miR390c | GhFH16 | 21 | 676 | 696 | AAGCUCAGGAGGGAUAGCGCC |
| ghr-miR479 | GhFH43 | 22 | 3701 | 3722 | CGUGAUAUUGGUUCGGCUCAUC |
| ghr-miR479 | GhFH11 | 22 | 2942 | 2963 | CGUGAUAUUGGUUCGGCUCAUC |
| ghr-miR479 | GhFH34 | 22 | 416 | 437 | CGUGAUAUUGGUUCGGCUCAUC |
| ghr-miR7484a | GhFH36 | 24 | 2599 | 2622 | UUUGUAUAUUAGAUCAAAGAGCAA |
| ghr-miR7484a | GhFH13 | 24 | 2584 | 2607 | UUUGUAUAUUAGAUCAAAGAGCAA |
| ghr-miR7484b | GhFH36 | 24 | 2599 | 2622 | UUUGUAUAUUAGAUCAAAGAGCAA |
| ghr-miR7484b | GhFH13 | 24 | 2584 | 2607 | UUUGUAUAUUAGAUCAAAGAGCAA |
| ghr-miR7491 | GhFH29 | 24 | 215 | 238 | UGGGAUCUUCGAGAGGAUUGAGCC |
| ghr-miR7491 | GhFH3 | 24 | 188 | 211 | UGGGAUCUUCGAGAGGAUUGAGCC |
| ghr-miR7495a | GhFH12 | 21 | 3956 | 3976 | UUACUUUAGAUGUCUCCUUCA |
| ghr-miR7495a | GhFH35 | 21 | 3062 | 3082 | UUACUUUAGAUGUCUCCUUCA |
| ghr-miR7495b | GhFH12 | 21 | 3956 | 3976 | UUACUUUAGAUGUCUCCUUCA |
| ghr-miR7495b | GhFH35 | 21 | 3062 | 3082 | UUACUUUAGAUGUCUCCUUCA |
| ghr-miR7500 | GhFH32 | 24 | 1238 | 1260 | AUCGAGUUAUUCGAGUUAAUCGAG |
| ghr-miR7502 | GhFH20 | 24 | 3870 | 3893 | UUUUUAACAGUAGAAAUGAAUGAA |
| ghr-miR7502 | GhFH43 | 24 | 4182 | 4205 | UUUUUAACAGUAGAAAUGAAUGAA |
| ghr-miR7505 | GhFH31 | 21 | 1210 | 1230 | UUCAGAAACCAUCCCUUCCUU |
| ghr-miR7508 | GhFH10 | 21 | 548 | 568 | CAAGAAAAGAAGUCGGGAGAG |
| ghr-miR7508 | GhFH33 | 21 | 548 | 568 | CAAGAAAAGAAGUCGGGAGAG |
| ghr-miR7509 | GhFH12 | 24 | 350 | 373 | UCAAAAGCACUUUUUGACAGCAAU |
| ghr-miR7510b | GhFH11 | 23 | 3257 | 3279 | AAGAACAUGAUCUUUAGCGGCGU |
| ghr-miR7510b | GhFH34 | 23 | 731 | 753 | AAGAACAUGAUCUUUAGCGGCGU |
| ghr-miR162a | GhFH26 | 21 | 2058 | 2078 | UCGAUAAACCUCUGCAUCCAG |
| ghr-miR167a | GhFH7 | 21 | 3188 | 3208 | UGAAGCUGCCAGCAUGAUCUA |
| ghr-miR167b | GhFH7 | 21 | 3188 | 3208 | UGAAGCUGCCAGCAUGAUCUA |
| ghr-miR2949a-3p | GhFH10 | 21 | 2639 | 2659 | UGCAAAUCCAGUCAAAAGUUA |
| ghr-miR2949a-3p | GhFH33 | 21 | 2651 | 2671 | UGCAAAUCCAGUCAAAAGUUA |
| ghr-miR2949b | GhFH42 | 22 | 2444 | 2465 | UCUUUUGAACUGGAUUUGCCGA |
| ghr-miR2949c | GhFH42 | 22 | 2444 | 2465 | UCUUUUGAACUGGAUUUGCCGA |
| ghr-miR2950 | GhFH13 | 21 | 344 | 364 | UGGUGUGCAGGGGGUGGAAUA |
| ghr-miR2950 | GhFH31 | 21 | 2520 | 2540 | UGGUGUGCAGGGGGUGGAAUA |
| ghr-miR2950 | GhFH36 | 21 | 573 | 593 | UGGUGUGCAGGGGGUGGAAUA |
| ghr-miR2950 | GhFH36 | 21 | 359 | 379 | UGGUGUGCAGGGGGUGGAAUA |
| ghr-miR3476-5p | GhFH23 | 21 | 2589 | 2609 | UGAACUGGGUUUGUUGGCUGC |
| ghr-miR3476-5p | GhFH46 | 21 | 2586 | 2606 | UGAACUGGGUUUGUUGGCUGC |
| ghr-miR396a | GhFH22 | 21 | 1855 | 1875 | UUCCACAGCUUUCUUGAACUG |
| ghr-miR396b | GhFH22 | 21 | 1855 | 1875 | UUCCACAGCUUUCUUGAACUG |
| ghr-miR399a | GhFH38 | 21 | 1573 | 1593 | CGCCAAUGGAGAUUUGUCCGG |
| ghr-miR399b | GhFH38 | 21 | 1573 | 1593 | CGCCAAUGGAGAUUUGUCCGG |
| ghr-miR399c | GhFH39 | 21 | 408 | 428 | UGCCAAAGGAGAGUUGGCCUU |
| ghr-miR399c | GhFH16 | 21 | 408 | 428 | UGCCAAAGGAGAGUUGGCCUU |
| ghr-miR479 | GhFH3 | 22 | 1301 | 1322 | CGUGAUAUUGGUUCGGCUCAUC |
| ghr-miR7484a | GhFH1 | 24 | 2635 | 2658 | UUUGUAUAUUAGAUCAAAGAGCAA |
| ghr-miR7484a | GhFH24 | 24 | 2632 | 2655 | UUUGUAUAUUAGAUCAAAGAGCAA |
| ghr-miR7484b | GhFH1 | 24 | 2635 | 2658 | UUUGUAUAUUAGAUCAAAGAGCAA |
| ghr-miR7484b | GhFH24 | 24 | 2632 | 2655 | UUUGUAUAUUAGAUCAAAGAGCAA |
| ghr-miR7485 | GhFH17 | 24 | 1890 | 1913 | AAAGACAUCUUUGAAUUCUUGGAG |
| ghr-miR7485 | GhFH17 | 24 | 1890 | 1913 | AAAGACAUCUUUGAAUUCUUGGAG |
| ghr-miR7485 | GhFH41 | 24 | 1887 | 1910 | AAAGACAUCUUUGAAUUCUUGGAG |
| ghr-miR7487 | GhFH11 | 24 | 1031 | 1054 | AUACUCUUAUAGGACACUUGUUAA |
| ghr-miR7488 | GhFH46 | 21 | 658 | 679 | UUUUGAGUACAGGG-GACAAAA |
| ghr-miR7491 | GhFH45 | 24 | 248 | 271 | UGGGAUCUUCGAGAGGAUUGAGCC |
| ghr-miR7491 | GhFH22 | 24 | 221 | 244 | UGGGAUCUUCGAGAGGAUUGAGCC |
| ghr-miR7493 | GhFH11 | 24 | 2855 | 2878 | AAUAUUUUAAUAAUUCAAUCGUCA |
| ghr-miR7494 | GhFH31 | 23 | 335 | 356 | AGCUUGUGGACUAGUUUUAACAA |
| ghr-miR7494 | GhFH7 | 23 | 464 | 485 | AGCUUGUGGACUAGUUUUAACAA |
| ghr-miR7494 | GhFH15 | 23 | 1298 | 1320 | AGCUUGUGGACUAGUUUUAACAA |
| ghr-miR7494 | GhFH38 | 23 | 1286 | 1308 | AGCUUGUGGACUAGUUUUAACAA |
| ghr-miR7495a | GhFH31 | 21 | 1260 | 1280 | UUACUUUAGAUGUCUCCUUCA |
| ghr-miR7495a | GhFH7 | 21 | 1260 | 1280 | UUACUUUAGAUGUCUCCUUCA |
| ghr-miR7495a | GhFH12 | 21 | 2081 | 2101 | UUACUUUAGAUGUCUCCUUCA |
| ghr-miR7495b | GhFH31 | 21 | 1260 | 1280 | UUACUUUAGAUGUCUCCUUCA |
| ghr-miR7495b | GhFH7 | 21 | 1260 | 1280 | UUACUUUAGAUGUCUCCUUCA |
| ghr-miR7495b | GhFH12 | 21 | 2081 | 2101 | UUACUUUAGAUGUCUCCUUCA |
| ghr-miR7499 | GhFH23 | 24 | 3485 | 3508 | AUAUAAUUUUCGGUUAAUUCGGUU |
| ghr-miR7499 | GhFH46 | 24 | 3482 | 3505 | AUAUAAUUUUCGGUUAAUUCGGUU |
| ghr-miR7500 | GhFH1 | 24 | 1563 | 1586 | AUCGAGUUAUUCGAGUUAAUCGAG |
| ghr-miR7500 | GhFH24 | 24 | 1560 | 1583 | AUCGAGUUAUUCGAGUUAAUCGAG |
| ghr-miR7501 | GhFH7 | 24 | 2628 | 2651 | AUAUCUGAUUCUGACACGAAAAAA |
| ghr-miR7502 | GhFH31 | 24 | 3612 | 3635 | UUUUUAACAGUAGAAAUGAAUGAA |
| ghr-miR7502 | GhFH7 | 24 | 3483 | 3506 | UUUUUAACAGUAGAAAUGAAUGAA |
| ghr-miR7502 | GhFH19 | 24 | 516 | 539 | UUUUUAACAGUAGAAAUGAAUGAA |
| ghr-miR7504a | GhFH23 | 24 | 2547 | 2570 | UAUGAAACUGUGAUUCCACGUCAU |
| ghr-miR7504a | GhFH46 | 24 | 2544 | 2567 | UAUGAAACUGUGAUUCCACGUCAU |
| ghr-miR7504b | GhFH17 | 24 | 1553 | 1575 | AGGAGGAAAAAUCUGAUUUGUCAU |
| ghr-miR7504b | GhFH17 | 24 | 1553 | 1575 | AGGAGGAAAAAUCUGAUUUGUCAU |
| ghr-miR7504b | GhFH41 | 24 | 1547 | 1569 | AGGAGGAAAAAUCUGAUUUGUCAU |
| ghr-miR7509 | GhFH31 | 24 | 221 | 244 | UCAAAAGCACUUUUUGACAGCAAU |
| ghr-miR7509 | GhFH7 | 24 | 350 | 373 | UCAAAAGCACUUUUUGACAGCAAU |
| ghr-miR7509 | GhFH2 | 24 | 1541 | 1564 | UCAAAAGCACUUUUUGACAGCAAU |
| ghr-miR7510b | GhFH5 | 23 | 874 | 896 | AAGAACAUGAUCUUUAGCGGCGU |
| ghr-miR7514 | GhFH37 | 24 | 502 | 525 | AUAAAGUGAUAAGUGAGAUCGUCU |
