# Supplementary figures and images for: Genome-wide identification and characterization of FORMIN gene family in cotton (Gossypium hirsutum L.) and their expression profiles in response to multiple abiotic stress treatments
Source: PLoS One. 2025 Mar 3;20(3):e0319176. doi: 10.1371/journal.pone.0319176 (PMC11875364; doi:10.1371/journal.pone.0319176)

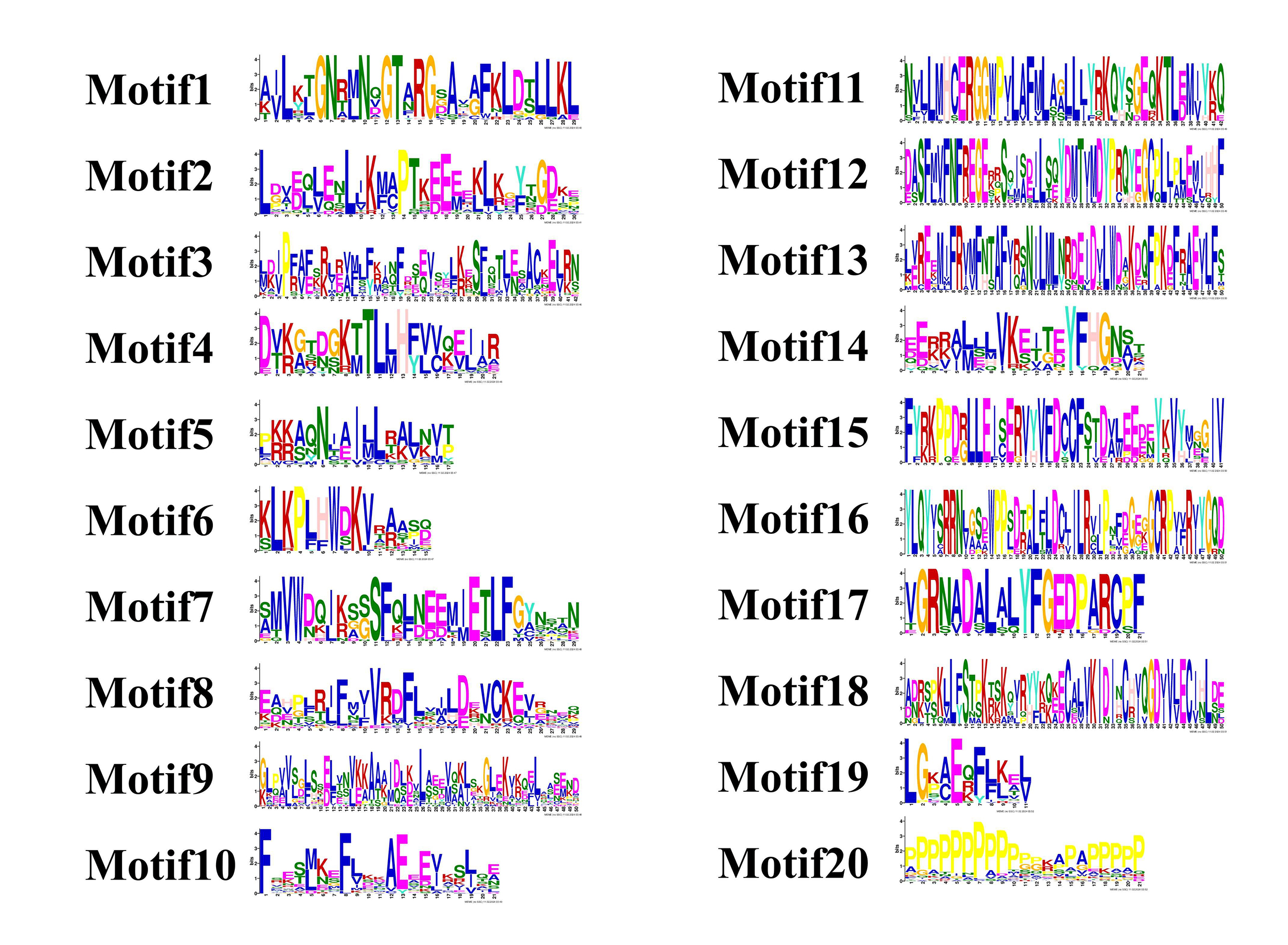

Supplement: S1 Fig — (TIF) [file pone.0319176.s016.tif]
